# Supplementary material for: A mixed methods study to develop a tool to assess institutional readiness to conduct knowledge translation activities in low-income and middle-income countries
Source: BMJ Open. 2021 Oct 10;11(10):e050049. doi: 10.1136/bmjopen-2021-050049 (PMC8506882; doi:10.1136/bmjopen-2021-050049)
Supplement: Supplementary data [file bmjopen-2021-050049supp001.pdf]

Appendix I: Readiness Questionnaire

Assessing and Enhancing the Readiness of Academic Institutions in LMICs to Conduct Knowledge Translation Activities

In what country is your institution based?
What is your current age?
Which gender best identifies you:

- Male
- Female
- Different identity
- Prefer not to answer

What is your current professional focus? (Select all that apply)

- Research
- Administration
- Leadership
- Project Coordination
- Communications
- External Affairs
- Development (e.g. Fundraising)
- Management
- Teaching
- Finance
- Information Technology
- Regulatory Services (e.g. IRB)
- Other (please describe) ______________________________

Do you do any KT work related to priority health areas in your country?

- Yes
- No
- Don't know

In your words, how do you define knowledge translation (KT)?

____

Kalbarczyk A, *et al. BMJ Open* 2022; 11:e050049. doi: 10.1136/bmjopen-2021-050049

Have you ever conducted knowledge translation (KT)?

Yes

No

Unsure

Have you conducted any of the following activities in relation to health issues in the past 3-5 years? (select all that apply)

Written a policy brief or prepared briefing notes

Written an evidence summary

Conducted a stakeholder meeting

Conducted a policy dialogue

Engaged with an advocacy campaign

Engaged with policy makers to set priorities

Developed a short video for a policy maker

Engaged with the media (e.g. journalists)

Used a knowledge translation platform

Authored or co-authored an article published in a peer-review journal

Conducted a systematic and/or rapid review

Taught a course on communication, advocacy, stakeholder engagement, or KT

Worked with a journalist to disseminate information

Given a presentation at a scientific conference

Knowledge Translation is also referred to as "knowledge to policy/practice", "knowledge exchange" and "evidence to action". If you're less familiar with KT, consider these terms as you complete the survey.

Kalbarczyk A, *et al. BMJ Open* 2022; 11:e050049. doi: 10.1136/bmjopen-2021-050049

Please identify the extent to which you agree or disagree with each of the following statements

	Strongly agree	Somewhat agree	Neither agree nor disagree	Somewhat disagree	Strongly disagree
I am confident that I can conduct KT activities.	○	○	○	○	○
People at my institution are confident they can conduct KT activities.	○	○	○	○	○
I feel personally motivated to do KT.	○	○	○	○	○
I feel personally motivated to do KT because I derive satisfaction and fulfillment.	○	○	○	○	○
I feel personally motivated to do KT because I receive rewards or benefits from my institution when I do.	○	○	○	○	○
I feel personally motivated to do KT because I will be punished by my institution if I do not.	○	○	○	○	○
Others in my institution feel motivated to do KT.	○	○	○	○	○

Kalbarczyk A, *et al. BMJ Open* 2022; 11:e050049. doi: 10.1136/bmjopen-2021-050049

Please identify the extent to which you agree or disagree with each of the following statements

	Strongly agree	Somewhat agree	Neither agree nor disagree	Somewhat disagree	Strongly disagree
I know what KT is.	○	○	○	○	○
I know how to do KT.	○	○	○	○	○
I have the skills to conduct KT.	○	○	○	○	○
I have experience conducting KT.	○	○	○	○	○
I have received training to conduct KT activities.	○	○	○	○	○
I frequently conduct KT activities.	○	○	○	○	○

What health areas or health topics have been the focus of your KT activities in the past 3-5 years? Please list up to 5.

○ Health Area/Topic 1 ________________________________

○ Health Area/Topic 2 ________________________________

○ Health Area/Topic 3 ________________________________

○ Health Area/Topic 4 ________________________________

○ Health Area/Topic 5 ________________________________

Kalbarczyk A, *et al. BMJ Open* 2022; 11:e050049. doi: 10.1136/bmjopen-2021-050049

Please identify the extent to which you agree or disagree with each of the following statements:

	Strongly agree	Somewhat agree	Neither agree nor disagree	Somewhat disagree	Strongly disagree
I am able to conduct KT in addition to my other tasks at this institution.	○	○	○	○	○
I have time to dedicate to KT in addition to my other tasks.	○	○	○	○	○
I believe conducting KT is an important part of my job.	○	○	○	○	○
People in my institution should conduct KT.	○	○	○	○	○
I am passionate about conducting KT.	○	○	○	○	○
I feel personally rewarded when I conduct KT.	○	○	○	○	○
I know hot to translate my data and key findings for policy makers.	○	○	○	○	○
KT activities have a positive impact on the health of communities.	○	○	○	○	○
Doing KT activities improves the national/local relevance of my institution.	○	○	○	○	○

Kalbarczyk A, *et al. BMJ Open* 2022; 11:e050049. doi: 10.1136/bmjopen-2021-050049

Please indicate the extent to which you agree or disagree with the following statements:

	Strongly agree	Somewhat agree	Neither agree nor disagree	Somewhat disagree	Strongly disagree
Senior leadership in my institution reward innovation and creativity in KT.	○	○	○	○	○
Senior Leadership in my institution provide staff with information on KT performance measures and guidelines.	○	○	○	○	○
In general in my institution, when there is agreement that KT needs to happen we have the necessary support in terms of budget or financial resources.	○	○	○	○	○
In general in my institution when there is agreement that KT needs to happen we have the necessary support in terms of training.	○	○	○	○	○
In general in my institution, when there is agreement that KT needs to happen we have the necessary support in terms of facilities.	○	○	○	○	○
In general in my institution when there is agreement that KT needs to happen we have the necessary support in terms of staffing.	○	○	○	○	○
Senior leadership management/staff opinion leaders at my institution set a high priority on the success of KT activities.	○	○	○	○	○
Senior leadership/clinical management in my institution reward innovation and creativity to improve KT.	○	○	○	○	○
Financial incentives are available for me to conduct KT (e.g. bonus salary).	○	○	○	○	○

My institution provides opportunities for professional development in KT (e.g. national and international conference support).	◯	◯	◯	◯	◯
My institution cares about improving health services for communities in my country.	◯	◯	◯	◯	◯
My colleagues believe it is important to translate our findings for policy makers and ministry members.	◯	◯	◯	◯	◯
Members of leadership believe it is important for me and my colleagues to translate findings for policy makers and ministry members.	◯	◯	◯	◯	◯
Conducting knowledge translation is an important consideration for promotion in my institution.	◯	◯	◯	◯	◯
My institution provides trainings on knowledge translation activities.	◯	◯	◯	◯	◯
If I want to conduct a KT activity, I know where to find people in my institution who can help.	◯	◯	◯	◯	◯
Senior members/leadership of my institution provide me with connections to conduct KT.	◯	◯	◯	◯	◯
People within my institution talk about their KT activities with each other.	◯	◯	◯	◯	◯

Kalbarczyk A, *et al. BMJ Open* 2022; 11:e050049. doi: 10.1136/bmjopen-2021-050049

My institution includes KT in its strategic plan, mission, or vision.

Strongly agree

Somewhat agree

Neither agree nor disagree

Somewhat disagree

Strongly disagree

If you indicated strongly agree or moderately/fairly agree to the question above, what health issues are prioritized by your institution in its strategic plan, mission or vision? (list up to 3)

○ Priority Health Issue 1 ____

○ Priority Health Issue 2 ____

○ Priority Health Issue 3 ____

Kalbarczyk A, *et al. BMJ Open* 2022; 11:e050049. doi: 10.1136/bmjopen-2021-050049

Please indicate the extent to which you agree or disagree with the following statements:

	Strongly agree	Somewhat agree	Neither agree nor disagree	Somewhat disagree	Strongly disagree
My institution is well connected to the ministry of health.	○	○	○	○	○
My institution determines research priorities in collaboration with the ministry of health.	○	○	○	○	○
If my institution does not conduct KT with the ministry, another college or university in my country will.	○	○	○	○	○
The funding organizations that support my research require KT activities.	○	○	○	○	○
The ministry relies on my institution more than other institutions to conduct KT.	○	○	○	○	○
Other institutions do more KT than my institution	○	○	○	○	○
Donors support KT activities because they want to have a real impact on health.	○	○	○	○	○
Ministry members and politicians in my country make health decisions without scientific consideration.	○	○	○	○	○
If ministry members demand my institution conducts KT activities it is because a donor or funder requires them to do so.	○	○	○	○	○
Members of my government understand the importance of scientific data for making decisions about health.	○	○	○	○	○
Members of my government want to work with my institution to improve health.	○	○	○	○	○
Most projects I am involved with have budgeted for communications and advocacy activities.	○	○	○	○	○

Kalbarczyk A, *et al. BMJ Open* 2022; 11:e050049. doi: 10.1136/bmjopen-2021-050049

Ministry members in my country prefer policy briefs to other forms of KT activities.	○	○	○	○	○

Please indicate the extent to which you agree or disagree with the following statements:

	Strongly agree	Somewhat agree	Neither agree nor disagree	Somewhat disagree	Strongly disagree
KT teams at my institution have clearly defined roles and responsibilities.	○	○	○	○	○
Other faculty and staff members are available to collaborate on KT activities.	○	○	○	○	○
I have regular opportunities to meet with ministry members to conduct KT.	○	○	○	○	○
Junior members of my team have opportunities to meet with ministry members to conduct KT.	○	○	○	○	○
I have at least one mentor who conducts KT with the ministry of health.	○	○	○	○	○
Senior members/Leadership of my institution use their networks to help others conduct KT.	○	○	○	○	○
I have time to regularly meet with members of the ministry to conduct KT activities.	○	○	○	○	○
I target my KT activities to different groups depending on the topic and who is involved.	○	○	○	○	○
Conducting KT activities is more of an art than a science.	○	○	○	○	○
My context plays a role in determining which KT activities I conduct.	○	○	○	○	○
Who I conduct KT activities with is as important as how I conduct the activities.	○	○	○	○	○
I spend a lot of time planning my KT activities.	○	○	○	○	○

Kalbarczyk A, *et al. BMJ Open* 2022; 11:e050049. doi: 10.1136/bmjopen-2021-050049

It is important to regularly meet with stakeholders when conducting KT activities.	○	○	○	○	○
KT activities conducted by my institution tend to be performed ad-hoc.	○	○	○	○	○
When conducting KT activities, it is important to engage a wide range of stakeholders.	○	○	○	○	○

Please indicate the extent to which you agree or disagree with each of the following statements:

	Strongly agree	Somewhat agree	Neither agree nor disagree	Somewhat disagree	Strongly disagree
The ministry of health requests KT activities from my institution.	○	○	○	○	○
When I conduct KT activities, they address current priorities of the ministry.	○	○	○	○	○
Financial resources are available at my institution to support the cost of KT.	○	○	○	○	○
KT activities require more resources than are available at my institution.	○	○	○	○	○
The research I conduct is determined by the priorities of international donors.	○	○	○	○	○
Financial resources are available at the ministry of health to support the cost of KT.	○	○	○	○	○
I am aware of donors that fund KT activities.	○	○	○	○	○

In your opinion, which of the following are the top three **facilitators** of KT at your institution?

Dedicated personnel

Funding

Protected Time

Training

Institutional mission/vision/strategy

Relationships with ministry members

Institutional rewards

Motivated faculty, staff, and leadership

Other (please describe) ____

In your opinion, which of the following are the top three **barriers** of KT at your institution?

Funding

Training

Time

Networks with the Ministry of Health and other stakeholders

Awareness of KT

Interest from policy makers

Understanding the policy process

Experience with KT

Financial incentives or rewards to conduct KT

Leadership

Other (please describe) ____

Kalbarczyk A, *et al. BMJ Open* 2022; 11:e050049. doi: 10.1136/bmjopen-2021-050049
