# Supplementary material for: A mixed methods study to develop a tool to assess institutional readiness to conduct knowledge translation activities in low-income and middle-income countries
Source: BMJ Open. 2021 Oct 10;11(10):e050049. doi: 10.1136/bmjopen-2021-050049 (PMC8506882; doi:10.1136/bmjopen-2021-050049)
Supplement: Supplementary data [file bmjopen-2021-050049supp002.pdf]

Appendix II: Survey questions (v1) with Median [IQR]

**Survey questions**	**Median [IQR]**
Q1 I am confident that I can conduct KT activities	3 [2-4]
Q2 People at my institution are confident they can conduct KT activities.	2 [2-4]
Q5 I feel personally motivated to do KT because I receive rewards or benefits from my institution when I do.	2 [1-4]
Q6 I feel personally motivated to do KT because I will be punished by my institution if I do not.	5 [2-5]
Q7 Others in my institution feel motivated to do KT.	2 [1-2]
Q8 I know what KT is.	2 [2-4]
Q11 I have experience conducting KT.	2 [2-4]
Q12 I have received training to conduct KT activities.	3 [2-5]
Q13 I frequently conduct KT activities.	3 [2-4]
Q15 I have time to dedicate to KT in addition to my other tasks.	2 [2-4]
Q17 People in my institution should conduct KT.	3 [2-3]
Q18 I am passionate about conducting KT.	2 [2-4]
Q20 I know how to translate my data and key findings for policy makers.	2 [2-3]
Q21 KT activities have a positive impact on the health of communities.	4 [2-4]
Q23 Senior leadership in my institution reward innovation and creativity in KT.	2 [1-3]
Q26 In general in my institution when there is agreement that KT needs to happen we have the necessary support in terms of training.	2 [1-3]
Q30 Senior leadership/clinical management in my institution rewards innovation and creativity to improve KT.	2 [1-3]
Q31 Financial incentives are available for me to conduct KT (e.g. bonus salary).	3 [2-5]
Q32 My institution provides opportunities for professional development in KT (e.g. national and international conference support).	2 [2-4]
Q33 My institution cares about improving health services for communities in my country.	4 [2-4]
Q36 Conducting knowledge translation is an important consideration for promotion in my institution.	2 [2-4]
Q37 My institution provides trainings on knowledge translation activities.	3 [1-3]
Q38 If I want to conduct a KT activity, I know where to find people in my institution who can help.	2 [2-3]
Q39 Senior members/leadership of my institution provide me with connections to conduct KT.	2 [2-3]
Q40 People within my institution talk about their KT activities with each other.	2 [1-3]
Q41 My institution includes KT in its strategic plan, mission, or vision.	2 [1-4]
Q42 My institution is well connected to the ministry of health.	2 [2-4]
Q43 My institution determines research priorities in collaboration with the ministry of health.	2 [2-4]
Q44 If my institution does not conduct KT with the ministry, another college or university in my country will.	2 [1-3]
Q45 The funding organizations that support my research require KT activities.	2 [1-3]
Q46 The ministry relies on my institution more than other institutions to conduct KT.	2 [1-3]
Q47 Other institutions do more KT than my institution.	1.5 [1-3]
Q48 Donors support KT activities because they want to have a real impact on health.	2 [2-4]

Q49 Ministry members and politicians in my country make health decisions without scientific consideration.	2 [2-4]
Q50 If ministry members demand my institution conducts KT activities it is because a donor or funder requires them to do so.	2 [1-4]
Q51 Members of my government understand the importance of scientific data for making decisions about health.	2 [2-4]
Q52 Members of my government want to work with my institution to improve health.	2 [2-3]
Q53 Most projects I am involved with have budgeted for communications and advocacy activities.	2 [1-3.5]
Q54 Ministry members in my country prefer policy briefs to other forms of KT activities.	2 [1-3]
Q55 KT teams at my institution have clearly defined roles and responsibilities.	2 [1-3]
Q56 Other faculty and staff members are available to collaborate on KT activities.	2 [1-3]
Q59 I have at least one mentor who conducts KT with the ministry of health.	3 [2-4]
Q60 Senior members/Leadership of my institution use their networks to help others conduct KT.	2 [2-4]
Q61 I have time to regularly meet with members of the ministry to conduct KT activities.	3 [2-4]
Q62 I target my KT activities to different groups depending on the topic and who is involved.	2 [2-3]
Q63 Conducting KT activities is more of an art than a science.	2 [2-4]
Q64 My context plays a role in determining which KT activities I conduct.	2 [2-4]
Q65 Who I conduct KT activities with is as important as how I conduct the activities.	2 [2-4]
Q66 I spend a lot of time planning my KT activities.	2 [1-3]
Q67 It is important to regularly meet with stakeholders when conducting KT activities.	3 [2-4]
Q68 KT activities conducted by my institution tend to be performed ad-hoc.	2 [1-2]
Q69 When conducting KT activities, it is important to engage a wide range of stakeholders.	4 [2-4]
Q70 The ministry of health requests KT activities from my institution.	2 [1-3]
Q71 When I conduct KT activities, they address current priorities of the ministry.	2 [1-3]
Q72 Financial resources are available at my institution to support the cost of KT.	2 [1-3]
Q73 KT activities require more resources than are available at my institution.	2 [2-4]
Q74 The research I conduct is determined by the priorities of international donors.	2 [2-4]
Q75 Financial resources are available at the ministry of health to support the cost of KT.	2 [1-3]
Q76 I am aware of donors that fund KT activities.	2 [1-3]
