# Supplementary material for: A mixed methods study to develop a tool to assess institutional readiness to conduct knowledge translation activities in low-income and middle-income countries
Source: BMJ Open. 2021 Oct 10;11(10):e050049. doi: 10.1136/bmjopen-2021-050049 (PMC8506882; doi:10.1136/bmjopen-2021-050049)
Supplement: Author's manuscript [file bmjopen-2021-050049.draft_revisions.pdf]

BMJ Open is committed to open peer review. As part of this commitment we make the peer review history of every article we publish publicly available.

When an article is published we post the peer reviewers' comments and the authors' responses online. We also post the versions of the paper that were used during peer review. These are the versions that the peer review comments apply to.

The versions of the paper that follow are the versions that were submitted during the peer review process. They are not the versions of record or the final published versions. They should not be cited or distributed as the published version of this manuscript.

BMJ Open is an open access journal and the full, final, typeset and author-corrected version of record of the manuscript is available on our site with no access controls, subscription charges or pay-per-view fees (http://bmjopen.bmj.com).

If you have any questions on BMJ Open's open peer review process please email info.bmjopen@bmj.com

BMJ Open

Developing a tool to assess institutional readiness to conduct knowledge translation activities in low-and middle-income countries

Journal:	*BMJ Open*
Manuscript ID	bmjopen-2021-050049
Article Type:	Original research
Date Submitted by the Author:	09-Feb-2021
Complete List of Authors:	Kalbarczyk, Anna; Johns Hopkins University Bloomberg School of Public Health, International Health<br>Rao, Aditi; Johns Hopkins University Bloomberg School of Public Health, International Health<br>Alonge, O; Johns Hopkins University Bloomberg School of Public Health
Keywords:	Health policy < HEALTH SERVICES ADMINISTRATION & MANAGEMENT, Organisational development < HEALTH SERVICES ADMINISTRATION & MANAGEMENT, PUBLIC HEALTH

SCHOLARONE™
Manuscripts

I, the Submitting Author has the right to grant and does grant on behalf of all authors of the Work (as defined in the below author licence), an exclusive licence and/or a non-exclusive licence for contributions from authors who are: i) UK Crown employees; ii) where BMJ has agreed a CC-BY licence shall apply, and/or iii) in accordance with the terms applicable for US Federal Government officers or employees acting as part of their official duties; on a worldwide, perpetual, irrevocable, royalty-free basis to BMJ Publishing Group Ltd ("BMJ") its licensees and where the relevant Journal is co-owned by BMJ to the co-owners of the Journal, to publish the Work in this journal and any other BMJ products and to exploit all rights, as set out in our licence.

The Submitting Author accepts and understands that any supply made under these terms is made by BMJ to the Submitting Author unless you are acting as an employee on behalf of your employer or a postgraduate student of an affiliated institution which is paying any applicable article publishing charge ("APC") for Open Access articles. Where the Submitting Author wishes to make the Work available on an Open Access basis (and intends to pay the relevant APC), the terms of reuse of such Open Access shall be governed by a Creative Commons licence – details of these licences and which Creative Commons licence will apply to this Work are set out in our licence referred to above.

Other than as permitted in any relevant BMJ Author's Self Archiving Policies, I confirm this Work has not been accepted for publication elsewhere, is not being considered for publication elsewhere and does not duplicate material already published. I confirm all authors consent to publication of this Work and authorise the granting of this licence.

For peer review only

Title: Developing a tool to assess institutional readiness to conduct knowledge translation activities in low-and middle-income countries

Authors: Anna Kalbarczyk, Aditi Rao, Olakunle O. Alonge

Institutional Address for Authors:

Johns Hopkins Bloomberg School of Public Health

615 N Wolfe St, Baltimore, MD 21205

Corresponding Author:

Anna Kalbarczyk, DrPH, MPH

Johns Hopkins Bloomberg School of Public Health

615 N Wolfe St, Baltimore, MD 21205

Email: akalbarc@jhu.edu

Phone: +1 410 502 9873

For peer review only

ABSTRACT:

Objective: Academic institutions are well-poised to conduct knowledge translation (KT) but face many barriers, particularly in low resource settings. Assessing the readiness of academic institutions to conduct KT can help our understanding of these barriers. However, limited research has been conducted to understand drivers of readiness for KT in low- and middle-income countries (LMICs) and there are no tools for KT readiness assessment designed for LMICs. This paper describes the development of a tool for assessing organizational readiness to conduct KT among academic institutions in LMICs.

Design: A literature review and stakeholder consultation process was conducted to identify constructs relevant for assessing KT readiness in LMICs. These were face validated with LMIC stakeholders and organized into Likert scale questionnaire. The questionnaire was distributed to researchers based at 6 LMIC academic institutions and members of a global knowledge-to-action thematic working group. An exploratory factor analysis was used to identify underlying dimensions for assessing institutional readiness to conduct KT.

Results: 111 respondents with varied KT experiences from 10 LMICs were included in the analysis. We selected five factors and 23 items, with factor loadings from 0.40 to 0.77. These factors include 1) institutional climate, 2) organization change efficacy, 3) prioritization and cosmopolitanism, 4) self-efficacy, and 5) financial resources. These factors accounted for 69% of the total variance, with Cronbach's alpha coefficients of 0.78, 0.73, 0.62, 0.68, and 0.52, respectively.

Conclusion: This study identifies a tool for assessing readiness of LMIC academic institutions to conduct KT, and unique opportunities for building capacity. The organizational focus of these

For peer review only

factors underscores the need for strategies that address organizational systems and structures in addition to individual skills. Future research will be conducted to understand determinants of these factors and develop a comprehensive set of capacity building strategies responsive to academic institutions in LMICs.

Keywords: knowledge translation, organizational readiness, factor analysis

Article Summary

<u>Strengths and Limitations of this study</u>

- The development of this tool was rooted in organizational change theory, building on validated tools.
- We conducted the research across multiple contexts in both Africa and Asia to capture constructs generalizable to institutions based in low-and middle-income countries.
- A significant limitation of this research is the small sample size. However, studies have argued smaller sample sizes may be justified when higher correlation coefficients are observed.

For peer review only - http://bmjopen.bmj.com/site/about/guidelines.xhtml

INTRODUCTION:

Evidence-based, cost-effective interventions designed to improve health are available but under-utilized, particularly among the poorest populations in low-and middle-income countries (LMICs) (1, 2). A recent Cochrane review identified a wide range of strategies for implementing such interventions in LMICs (3). This gap between what we know through research and what is done in practice (i.e. 'know-do' gap), underscores the need for improved utilization of research by policy makers and practitioners in LMICs.

Knowledge Translation (KT) is one approach to addressing this gap that describes a process of research generation, synthesis, sharing, and utilization to improve health. Many definitions for KT exist and we use the one developed by the Canadian Institute of Health Research: "A dynamic and iterative process that includes the synthesis, dissemination, exchange, and ethically sound application of knowledge to improve health, provide more effective health services, and strengthen the health care system" (4). Examples of KT activities include evidence synthesis via policy briefs and systematic reviews, sharing knowledge through communities of practice, and forming large-scale partnerships and networks, which can been applied at different policy and practice levels to facilitate uptake of evidence and improve population health (5).

Academic institutions in LMICs are well-positioned to conduct KT activities as respected producers of research evidence and their ability to serve as knowledge brokers, but face a unique set of barriers driven by contextual factors including structural inequities (e.g. historically imbalanced relationships with institutions in the global north and research agendas driven by outside researchers) (6, 7) and limited resources (8). We conducted a qualitative study with participants in academic institutions in 6 countries (Bangladesh, Democratic Republic of the Congo, Ethiopia, India, Indonesia, and Nigeria) and found that soft-skills (i.e. communication,

For peer review only

self-awareness, and adaptability), robust networks, and alignment between institutional priorities and incentives are important factors that shape institutional capacity to conduct KT activities in LMICs (9). While strategies such as trainings, mentorship, and institutional leadership engagement have been developed to address these barriers and specific factors (10, 11), these strategies have been mostly applied to academic institutions in high-income countries (HICs) (12-14) with limited empirical evidence of their effectiveness in LMIC settings (15). Irrespective of the strategies enacted to address barriers to conducting KT activities, academic institutions need to be self-aware on their role in KT, and readiness to undertake relevant KT activities addressing priority health issues to be successful (16).

Indeed, some institutions are becoming increasingly aware of the importance of organizational readiness and the need to implement processes that motivate and enhance change to facilitate the conduct of KT activities or address barriers to conducting KT activities (17, 18). Such processes could be motivated by increased demand from policy makers for evidence-based data or funders offering new opportunities that prioritize or require KT activities. Whereas specific KT activities (e.g., systematic review, establishing a community of practice) may vary for different academic institutions given differences in their priority health issue focus, policy cycle and resources – the similarities in the structure, function and objectives of academic institutions provide a conceptual basis for assessing their readiness to conduct different KT activities across different settings. Assessing organizational readiness enables exploration of facilitators and barriers for individuals and organizations to implement capacity building strategies (19), and has been used to tailor approaches for addressing barriers to KT activities (20). However, most of these readiness assessments have been conducted in HICs, and there is an underuse of validated tools (18).

For peer review only - http://bmjopen.bmj.com/site/about/guidelines.xhtml

Validated readiness tools, used to assess institutional readiness for change (e.g. to implement any intervention, including KT activities), include Organizational Readiness for Implementing Change (ORIC) (19), Organizational Readiness to Change Assessment (ORCA) (21) and Texas Christian University, Organizational Readiness to Change (TCU ORC) (22). These tools largely overlap and assess domains such as availability of resources, individual attributes and motivation, and organizational climate (19, 21, 22). Psychometric assessments have been conducted on these tools which largely support their reliability and structure (19, 23, 24). Most tools have been implemented in HICs, largely in health care settings including hospitals, primary care settings, public health agencies, and public sector organizations, and not specifically for KT. Gagnon et al. developed the OR4KT tool, designed to assess organizational readiness for KT in healthcare organizations, but they note that its applicability to LMICs may be limited given how different healthcare organizations are organized comparing HIC to LMIC settings (20). Our literature review identified only one validated tool, the TCU ORC, that had been used in a LMIC (25). This study utilized the TCU model to examine the organizational functioning of drug treatment facilities in South Africa but makes no mention of adapting the tool to address unique contextual factors. A recent systematic review identified 30 tools designed to address organizational readiness for institutions in LMICs but none were validated (26). The lack of readiness assessments specific to KT and availability of adapted tools for conducting readiness assessment further contributes to the 'know-do' gap in LMICs.

This paper describes an empirical study to develop a tool for assessing institutional readiness to conduct KT among academic institutions in LMICs. The paper builds on published tools for readiness assessments more broadly and developed for HIC settings and identifies constructs that

For peer review only - http://bmjopen.bmj.com/site/about/guidelines.xhtml

are relevant for assessing readiness of academic institutions to do KT activities in LMICs, and the validity and reliability of an adapted tool measuring these constructs for LMIC contexts.

METHODS:

This research is embedded in a five-year parent-project, "Synthesis and Translation of Research and Innovations from Polio Eradication" (STRIPE), designed to map, synthesize, and disseminate knowledge from global efforts to eradicate polio (27). The STRIPE consortium is led by Johns Hopkins University in collaboration with 6 academic institutions and 1 research consultancy firm based in LMICs. This research engaged the 6 academic collaborators, shown in **Table 1**, and was conducted in 3 sequential steps:

Table 1: STRIPE Academic Partners

Institution Name	Country
James P Grant School of Public Health, BRAC University	Bangladesh
School of Public Health, University of Kinshasa	Democratic Republic of the Congo (DRC)
College of Health Sciences, Addis Ababa University	Ethiopia
Indian Institute of Health Management Research University	India
Faculty of Medicine, Public Health, and Nursing, Gadjah Mada University	Indonesia
College of Medicine, University of Ibadan	Nigeria

Step 1: A Literature review was conducted to identify validated tools for measuring individual and institutional readiness. Standard search tools including PubMed and Google Scholar were searched in 2019 using combinations of the following terms: "tools", "organizational readiness", and "institutional assessments". Thematic constructs and questionnaire items from the identified tools were extracted and organized by common domains. These were then mapped to the domains and constructs of the Consolidated Framework for Implementation Research (CFIR) which helped identify important gaps for implementation in LMICs. CFIR, a well-known

For peer review only - http://bmjopen.bmj.com/site/about/guidelines.xhtml

framework, is comprised of five domains including intervention characteristics, outer setting, inner setting, individual characteristics, and implementation process (28). The framework has been widely used in LMICs as a practical structure for approaching complex programmatic and policy challenges (from planning to implementation to evaluation) such as conducting KT activities (29).

Institutional data was also collected from each academic partner including strategic plans, organograms, and information on recently published articles. Public information from each country's Ministry of Health (MoH) including recent reports and strategic plans were collected. Using the institutional data and information from the MoH, a content analysis was performed to establish health reference points for participants from each country (i.e. specific priority health issues on which the academic institution and ministry of health align).

Step 2: We conducted a **stakeholder consultation** process to evaluate the face and content validity of the constructs and items described from the review phase for LMIC contexts and explore new themes. The 18 stakeholders included representatives from each of the 6 academic STRIPE institutions in addition to their external government partners. Internal representatives met at least one of the following criteria: 1) Individuals involved in institutionally prioritized KT activities (health-issue specific), 2) Individuals involved in making strategic/policy decisions around KT activities at the institutional level, 3) Individuals in leadership that determine the internal context and external relationships of the institutions. External stakeholders included policy makers who currently engaged or had been engaged with the academic institution in the past two years in efforts to conduct KT activities.

Inputs from this process focused largely on providing feedback on constructs of readiness as they related to less well-operationalized domains and constructs of CFIR in the literature (e.g. outer

For peer review only - http://bmjopen.bmj.com/site/about/guidelines.xhtml

setting and characteristics of individuals). This process was also used to confirm health priorities in each country and develop a list of KT activities commonly conducted across the settings. A draft tool for readiness assessment was then developed based on findings from the review and consultation steps. Country-level priorities and activities were incorporated throughout the tool to help illustrate complex KT items for participants.

Step 3: We developed a **quantitative survey** to assess the psychometric properties of the draft tool.

Tool design: The draft tool from step 2 was converted to a questionnaire using Microsoft Excel ©. Questions were grouped by demographics, the five CFIR domains, and barriers and facilitators to KT.

Demographic questions included country, gender, age, past experience with KT, and professional focus. Items designed to assess readiness were developed on a 5-point Likert scale ('strongly disagree to strongly agree'), following the design of the reviewed validated readiness tools. The final questions asked participants to select the top three barriers and facilitators to KT activities from a list generated based on steps 1 and 2. Negatively worded questions were reversed in numeric value, so the number 5 consistently reflected positive attitudes.

We followed guidelines outlined by Choi and Pak to avoid questionnaire biases (30). This included refraining from irrelevant redundancies, incorporating largely positively worded items, and utilizing priority references and activities to avoid ambiguity. Open-ended questions were minimized to reduce response fatigue and the format was designed to cluster Likert-scale questions, reducing the length of the questionnaire.

For peer review only - http://bmjopen.bmj.com/site/about/guidelines.xhtml

Survey population and procedure: The study population for the survey included faculty, staff, and leadership at each of the 6 academic STRIPE institutions in addition to members of the Translating Evidence to Action Working Group (TWG) within the Health Systems Global consortium who currently work for academic institutions based in LMICs. The TWG is a multidisciplinary group comprised of approximately 220 researchers, decision-makers, and implementers with a focus on the translation of health systems evidence into action (that is, KT) and supporting mechanisms to share best practices globally. STRIPE Principal Investigators (PI) at each institution identified faculty, staff, and leadership who had been involved in KT at their institution, amongst whom the survey was then distributed. The eligibility criteria for participating in the survey included: 1) must be an active member (e.g. staff, faculty, leadership) of an academic institution within the STRIPE consortium or TWG and 2) your institution must be based in a LMIC. A total of 200 eligible participants were identified via the STRIPE consortium - and were invited to participate in the survey via Qualtrics ©, an online survey software, with options to respond in English or French. TWG does not track demographics on its participants so we do not know, of the 220 members, how many were eligible to complete the survey.

The survey questions were the same for all participants. However, the health priority reference points and list of KT activities identified for each institution in Step 2 was used to illustrate the survey questions, and these differed from one academic institution to another. Members of the TWG, all highly familiar with KT, were not provided health priority references but were asked which health issues their institutions prioritized. The links to the Qualtrics surveys were circulated via email through STRIPE PIs, members of their leadership, or through local research

For peer review only - http://bmjopen.bmj.com/site/about/guidelines.xhtml

assistants. Members of the thematic working group were contacted via email using the group's listserv. Survey data was collected from February 06, 2020 – March 25, 2020.

Responses were monitored for completeness (missing values) and correctness (invalid responses, outliers, and extreme observations). In-country liaisons at each STRIPE institution were asked to follow-up with potential respondents who may have started the survey but not completed it.

Analysis: We conducted Exploratory Factor Analysis (EFA) to identify underlying dimensions of institutional capacity and readiness to conduct KT activities among LMIC institutions. The process was iterative and exploratory, wherein each observed variable was considered as being a potential measure of factors, and the goal being to determine relationships between observed variables and factors that are strongest. EFA encompasses completing a series of sequential steps that involves evaluation of various options and making a decision at each step. These include assessing sample size adequacy, requisite correlation between variables, appropriate number of factors to include in the model, as well as selection of the factor rotation method.

We began with a sample size (N) to items (p) ratio of approximately 1.5:1. Guiding norms on an adequate sample size for conducting EFA state a desired ratio of at least 5:1 (31), while others agree smaller sample sizes can be justified when higher correlation coefficients are present (32). Adequacy of sample size was assessed via the Kaiser-Meyer-Olkin (KMO) test. The KMO statistic indicates the proportion of variance in the variables that might be cause by underlying factors. A score above 0.5 is generally regarded as acceptable. We proceeded with a Pearson bivariate correlation of all items, inspecting for markedly high values, which indicate multicollinearity. Ultimately for pairs with correlation coefficients greater than 0.5, one item from each pair was removed based on a qualitative analysis of the variables. Factorability was confirmed by Bartlett's test of sphericity, which tests the null hypothesis that the original

For peer review only - http://bmjopen.bmj.com/site/about/guidelines.xhtml

correlation matrix is an identity matrix, indicating that variables are unrelated and therefore unsuitable for structure detection. A significant value (<0.05) is requisite. Multicollinearity was explored via the determinant of the matrix, which should be greater than 0.00001.

The survey questions then underwent factor analysis (FA) with an oblique rotation (oblimin). We chose FA over principal component analysis, which assumes the total variance of the variables can be accounted for by means of its components (or factors), and hence that there is no error variance. Alternatively, FA assumes that the variables do not account for 100% of the variance, allowing it to be more flexible. Subsequently, a factor rotation allowed us to obtain a simpler, more interpretable solution for the FA. The choice of rotation depends on whether there is good theoretical reason to suppose the factors should be related or independent of each other. An oblique rotation allows a degree of correlation between the factors. In assessing the number of factors to retain, we considered multiple criteria. Factors with eigenvalues less than 1 were excluded, based on Guttman-Kaiser's rule. Using a Scree plot, all factors after the elbow were excluded. Only items that loaded highly (>0.5) and uniquely on each factor were retained. Factors with fewer than three variables loading on to it were also excluded. The proportion of the total variance explained by the retained factors was examined to be above the recommended minimum of 50%. Finally, Cronbach's alpha coefficient was calculated to test for scale reliability and internal consistency within and between factors. The norm is to accept values 0.7 or higher.

Data analysis was conducted in Stata version 13 (33).

This research was declared "Non-Human Subjects" by the Johns Hopkins Bloomberg School of Public Health Institutional Review Board on October 08, 2019.

For peer review only - http://bmjopen.bmj.com/site/about/guidelines.xhtml

RESULTS:

Review of the literature revealed 26 organizational readiness tools related to health institutions (including hospitals, clinics, research institutes, etc.) and 30 tools developed specifically to assess organizational readiness for implementing global health interventions; only 1 was designed specifically for KT. Both sets of tools had been collated and analyzed in systematic reviews (23, 26). Common constructs across these tools included availability of resources, individual attributes and motivation, and organizational climate.

Eighteen stakeholders, both internal and external to the 6 STRIPE academic institution were interviewed. Three cross-cutting themes emerged as relevant for readiness to conduct KT in LMICs. This included 1) the complexity of the policy process and necessity of "soft-skills" (i.e., communication, self-awareness, relationship maintenance), 2) misalignment between institutional missions and incentives and 3) the role of internal and external networks. The results from the consultation process are described in more detail elsewhere (9). The constructs identified from the literature review and the cross-cutting themes from the consultation process were organized into a quantitative tool with 5 domains and 76 items, with new items developed for the themes. A total of 9 additional questions on demographics, facilitators, and barriers to doing KT were added to translate the tool into a survey questionnaire [Appendix 1].

We received 158 responses to the survey across the 6 institutions and TWG. There were 47 respondents who completed 9% or less of the survey, which were subsequently dropped from analysis. These respondents did not cluster by country, age, or gender and appeared random. A total of 111 responses were included in the final analysis. According to Arrindell and van der Ende, a sample size (N) to items (p) ratio, i.e. N:p ratio of 3:1 is adequate for demonstrating a

For peer review only - http://bmjopen.bmj.com/site/about/guidelines.xhtml

stable factor structure with an alpha level of 0.05 (32). Hence, our sample size of 111 is adequate for demonstrating the validity and reliability of a tool with at least 37 items.

There were 27 respondents from Bangladesh, 19 from Indonesia, 16 from India, 16 from Nigeria, 12 from DRC, 11 from Ethiopia, and 10 from the TWG representing other LMICs. Of the total 111 respondents, 53% (n=59) of respondents were male and 43% (n=48) were female. A majority of respondents (57%, n=64) were 30-49 years of age and most (59%, n=66) indicated they had experience conducting KT. Participants were asked to indicate their current professional focus/foci. Most respondents were engaged in research (83%, n=93) and teaching (62%, n=69). Other common foci included project coordination (36%, n=41), leadership (15%, n=17), and management (16%, n=18).

The most commonly conducted KT activities included "taught a course on communication, advocacy, stakeholder engagement or KT" (98%, n=109), "conducted a stakeholder meeting" (62%, n=69), and "given a presentation at a scientific conference" (60%, n=67). Individuals who indicated having experience with KT activities, compared to those with no experience, were significantly more likely to have written a policy brief (p-value 0.0059), conducted a stakeholder meeting (p-value 0.0364), engaged with policy makers to set priorities (p-value 0.0129), and to have given a presentation at a scientific conference (p-value 0.0011). Two KT activities also varied significantly by country, "authored or co-authored an article in a peer-review journal" (p-value 0.0001), and "given a presentation at a scientific conference" (p-value 0.0002). Additional descriptive statistics are presented in **Table 2**.

For peer review only - http://bmjopen.bmj.com/site/about/guidelines.xhtml

Table 2: Characteristics of Respondents

Variable	Bangladesh n=27 (%)	DRC n=12 (%)	Ethiopia n=11 (%)	India n=16 (%)	Indonesia n=19 (%)	Nigeria n=16 (%)	Others n=10 (%)	Total n=111 (%)
**Age**								
18-29 years	13 (48.15)	0 (0.00)	0 (0.00)	0 (0.00)	6 (31.58)	0 (0.00)	0 (0.00)	19 (17.12)
30-49 years	12 (44.44)	6 (50.00)	8 (72.73)	7 (43.75)	11 (57.89)	12 (75.00)	8 (80.00)	64 (57.66)
50-69 years	2 (7.41)	5 (41.67)	3 (27.27)	7 (43.75)	0 (0.00)	3 (18.75)	2 (20.00)	22 (19.82)
≥70 years	0 (0.00)	1 (8.33)	0 (0.00)	0 (0.00)	0 (0.00)	0 (0.00)	0 (0.00)	1 (0.90)
**Gender**								
Male	16 (59.26)	6 (50.00)	8 (72.73)	10 (62.50)	5 (26.32)	9 (56.25)	5 (50.00)	59 (53.15)
Female	9 (33.33)	5 (41.67)	3 (27.27)	6 (37.50)	14 (73.68)	7 (43.75)	4 (40.00)	48 (43.24)
**Professional Focus**								
Research	22 (81.48)	12 (100.00)	10 (90.91)	14 (87.50)	14 (73.68)	13 (81.25)	8 (80.00)	93 (83.78)
Administration	1 (3.70)	1 (8.33)	0 (0.00)	1 (6.25)	1 (5.26)	5 (31.25)	0 (0.00)	9 (8.11)
Leadership	1 (3.70)	1 (8.33)	0 (0.00)	4 (25.00)	3 (15.79)	6 (37.50)	2 (20.00)	17 (15.32)
Project Coordination	6 (22.22)	6 (50.00)	1 (9.09)	12 (75.00)	8 (42.11)	5 (31.25)	3 (30.00)	41 (36.94)
Communications	1 (3.70)	0 (0.00)	0 (0.00)	3 (18.75)	1 (5.26)	1 (6.25)	2 (20.00)	8 (7.21)
External Affairs	0 (0.00)	2 (16.67)	0 (0.00)	0 (0.00)	1 (5.26)	0 (0.00)	0 (0.00)	3 (2.70)
Development	0 (0.00)	0 (0.00)	0 (0.00)	3 (18.75)	1 (5.26)	1 (6.25)	1 (10.00)	6 (5.41)
Management	4 (14.81)	1 (8.33)	0 (0.00)	6 (37.50)	3 (15.79)	3 (18.75)	1 (10.00)	18 (16.22)

For peer review only - http://bmjopen.bmj.com/site/about/guidelines.xhtml

For peer review only

Teaching	7 (25.93)	12 (100.00)	10 (90.91)	14 (87.50)	4 (21.05)	15 (93.75)	7 (70.00)	69 (62.16)
Finance	0 (0.00)	0 (0.00)	0 (0.00)	0 (0.00)	0 (0.00)	0 (0.00)	0 (0.00)	0 (0.00)
IT	0 (0.00)	0 (0.00)	0 (0.00)	1 (6.25)	0 (0.00)	0 (0.00)	0 (0.00)	1 (0.90)
Regulatory Services	2 (7.41)	1 (8.33)	0 (0.00)	1 (6.25)	0 (0.00)	0 (0.00)	1 (10.00)	5 (4.50)
**KT Experience**								
Yes	14 (51.85)	5 (41.67)	7 (63.64)	14 (87.50)	12 (63.16)	11 (68.75)	3 (30.00)	66 (59.46)
No	9 (33.33)	1 (8.33)	1 (9.09)	1 (6.25)	2 (10.53)	4 (25.00)	3 (30.00)	21 (18.92)
Unsure	4 (14.81)	6 (50.00)	3 (27.27)	1 (6.25)	4 (21.05)	1 (6.25)	3 (30.00)	22 (19.82)
**KT Activities**								
Written a policy brief	10 (37.04)	3 (25.000	2 (18.18)	8 (50.00)	11 (57.89)	7 (43.75)	3 (30.00)	44 (39.64)
Written an evidence summary	5 (18.52)	4 (33.33)	4 (36.36)	7 (43.75)	7 (36.84)	5 (31.25)	3 (30.00)	35 (31.53)
Conducted a stakeholder meeting	12 (44.44)	7 (58.33)	5 (45.45)	12 (75.00)	16 (84.21)	10 (62.50)	7 (70.00)	69 (62.16)
Conducted a policy dialogue	3 (11.11)	1 (8.33)	0 (0.00)	6 (37.50)	5 (26.32)	2 (12.50)	2 (20.00)	19 (17.12)
Engaged with an advocacy campaign	5 (18.52)	3 (25.00)	1 (0.09)	7 (43.75)	6 (31.58)	7 (43.75)	2 (20.00)	31 (27.93)
Engaged with policy makers to set priorities	3 (11.11)	4 (33.33)	3 (27.27)	6 (37.50)	9 (47.37)	5 (31.25)	4 (40.00)	34 (30.63)
Developed a video for a policy maker	5 (18.52)	1 (8.33)	0 (0.00)	4 (25.00)	4 (21.05)	2 (12.50)	1 (10.00)	17 (15.32)
Engaged with the media	5 (18.52)	3 (25.00)	2 (18.18)	8 (50.00)	5 (26.32)	8 (50.00)	5 (50.00)	36 (32.43)
Used a KT platform	4 (14.81)	4 (33.33)	2 (18.18)	7 (43.75)	4 (21.05)	0 (0.00)	3 (30.00)	24 (21.62)
Authored or co-authored	8 (29.63)	9 (75.00)	7 (63.64)	11 (68.75)	7 (36.84)	16 (100.00)	9 (90.00)	67 (60.36)
Conducted a systematic or rapid review	6 (22.22)	1 (8.33)	6 (54.55)	5 (31.25)	2 (10.53)	4 (25.00)	4 (40.00)	28 (25.23)
Taught a course on communication, advocacy, stakeholder engagement, or	27 (100.00)	12 (100.00)	11 (100.00)	14 (87.50)	19 (100.00)	16 (100.00)	10 (100.00)	109 (98.20)

For peer review only - http://bmjopen.bmj.com/site/about/guidelines.xhtml

For peer review only

KT								
Worked with a journalist to disseminate information	2 (7.41)	3 (25.00)	0 (0.00)	6 (37.50)	3 (15.79)	4 (25.00)	2 (20.00)	20 (18.02)
Given a presentation at a scientific conference	8 (29.63)	7 (58.33)	6 (54.55)	14 (87.50)	9 (47.37)	15 (93.75)	8 (80.00)	67 (60.36)

† Data were missing for some variables, therefore numbers do not always add to the total.

For peer review only

We ran an exploratory factor analysis on the complete data set (version 0) which included 76 items. This approach yielded 22 factors; both the KMO measure and Barlett's test yielded no value. Constructs covered in these factors included individual motivation, organizational climate, organizational culture, internal resources, individual knowledge and skills, internal and external networks, funding sources, prioritization, and shared ethos for change (change valence). Many factors overlapped, each addressing similar or related constructs. The correlation matrix was reviewed for highly correlated items and those with correlations above 0.5 were dropped; 17 items were removed in this process. Highly correlated items included, "Q1: I am confident that I can conduct KT activities", "Q3: I feel personally motivated to do KT", and "Q10 I have the skills to conduct KT". Q10 was also heavily correlated with "Q9: I know how to do KT" and "Q11: I have experience conducting KT". Wherever possible, items were kept that did not correlate heavily with other items. These items were also reviewed to ensure they captured the same or similar information.

For the remaining 59 items (version 1.0) we repeated the EFA, followed by an oblique rotation, producing as simple a structure as possible while permitting correlations among factors. This yielded 17 factors, with a KMO measure of 0.4 and a significant value for Bartlett's test (p=0.000). The items, their medians, and inter-quartile range (IQR) for version 1.0 can be found in the supplemental material [Appendix 2]. Individual motivation, networks, prioritization, organizational climate, and resources were still captured by these factors. New constructs emerged in this model including institutional peer pressure, the process of conducting KT, and perceived value of KT.

All cross-loading items (i.e. items loading on more than one factor) or with a loading less than 0.5 were then dropped, resulting in the removal of 26 additional items (version 2.0). **Table 3**

For peer review only - http://bmjopen.bmj.com/site/about/guidelines.xhtml

shows each item included in version 2.0, the item's median, and IQR. Some dropped items addressed individual motivation (e.g. Q13: I am passionate about conducting KT" and "KT activities have a positive impact on the health of communities") and institutional climate (e.g. Q32: My institution provides opportunities for professional development in KT"; Q59: I have at least one mentor who conducts KT with the ministry of health"). We re-ran the factor analysis with 31 items, followed by oblique rotation, and identified seven factors. The model produced a KMO of 0.52 and a significant value for Barlett's test (p=0.000). In this model one item (Q38: If I want to conduct a KT activity, I know where to find people in my institution who can help) loaded on two factors and was thus removed; in the resulting model, another item loaded to 2 factors and the item was dropped. A final set of 31 items was retained.

Table 3: Survey questions (v2) with Median [IQR]

**Survey questions**	**Median [IQR]**
Q26 In general in my institution when there is agreement that KT needs to happen we have the necessary support in terms of training.	3 [2-4]
Q30 Senior leadership/clinical management in my institution rewards innovation and creativity to improve KT.	2 [1-3]
Q31 Financial incentives are available for me to conduct KT (e.g. bonus salary).	3 [2-5]
Q37 My institution provides trainings on knowledge translation activities.	3 [1-3]
Q38 If I want to conduct a KT activity, I know where to find people in my institution who can help.	4 [3-4]
Q39 Senior members/leadership of my institution provide me with connections to conduct KT.	2 [2-3]
Q40 People within my institution talk about their KT activities with each other.	2 [1-3]
Q56 Other faculty and staff members are available to collaborate on KT activities.	2 [1-3]
Q2 People at my institution are confident they can conduct KT activities.	2 [2-4]
Q6 I feel personally motivated to do KT because I will be punished by my institution if I do not.	5 [3-5]
Q7 Others in my institution feel motivated to do KT.	2 [1-2]
Q44 If my institution does not conduct KT with the ministry, another college or university in my country will.	4 [3-4]
Q45 The funding organizations that support my research require KT activities.	4 [3-4]

For peer review only - http://bmjopen.bmj.com/site/about/guidelines.xhtml

Q46 The ministry relies on my institution more than other institutions to conduct KT.	2 [1-3]
Q47 Other institutions do more KT than my institution.	3 [2-3]
Q51 Members of my government understand the importance of scientific data for making decisions about health.	2 [2-4]
Q52 Members of my government want to work with my institution to improve health.	4 [3-4]
Q54 Ministry members in my country prefer policy briefs to other forms of KT activities.	4 [3-4]
Q69 When conducting KT activities, it is important to engage a wide range of stakeholders.	4 [2-4]
Q41 My institution includes KT in its strategic plan, mission, or vision.	2 [1-4]
Q49 Ministry members and politicians in my country make health decisions without scientific consideration.	2 [2-4]
Q60 Senior members/Leadership of my institution use their networks to help others conduct KT.	2 [2-4]
Q66 I spend a lot of time planning my KT activities.	2 [1-3]
Q71 When I conduct KT activities, they address current priorities of the ministry.	2 [1-3]
Q76 I am aware of donors that fund KT activities.	3 [2-4]
Q11 I have experience conducting KT.	4 [3-4]
Q12 I have received training to conduct KT activities.	3 [2-5]
Q15 I have time to dedicate to KT in addition to my other tasks.	2 [2-4]
Q20 I know how to translate my data and key findings for policy makers.	2 [2-3]
Q55 KT teams at my institution have clearly defined roles and responsibilities.	2 [1-3]
Q53 Most projects I am involved with have budgeted for communications and advocacy activities.	2 [1-3.5]
Q63 Conducting KT activities is more of an art than a science.	2 [2-4]
Q75 Financial resources are available at the ministry of health to support the cost of KT.	2 [1-3]

We re-ran the FA with the 31 retained items to identify a five-factor model. Items were selected based on the strength of the factor loading, uniqueness of the factors, the resulting scree-plot, and cross-loading criteria – and we selected 23 items for the five factors, with a N:p ratio of 5:1. **Figure 1** displays the eigen value plot of the final factor model. For the final five factor model, the average communality (aka uniqueness) of selected items was 0.61. The items (p) per factor (r) ratio (p:r ratio) in the final model presented in Table 3 is 23:5 with N = 111. According to MacCallum et al., sample sizes of N = 100 and N = 200 are needed to estimate stable factor structure with 95% convergence for p:r ratio of 20:3 and 10:3 respectively if the average

For peer review only - http://bmjopen.bmj.com/site/about/guidelines.xhtml

communality is low (less than 0.4) (31). If the communality is high (>0.4), as found in our study, a N = 60 is adequate for p:r ratio of 10:3 or 20:3 and will estimate the factor structure with over 99% convergence.

[Insert Figure 1]

The five final factors that emerged from the analysis were named 1) Institutional Climate, 2) Organization Change Efficacy, 3) Prioritization and Cosmopolitanism, 4) Self-efficacy, and 5) Financial resources, based on their item characteristics and the underlying theories for those items (9). Factor 1 contains 6 items and was labeled 'institutional climate' because each item described aspects of their institution, colleagues, and leadership. Factor 2 contains 5 items and was labeled 'organization change efficacy' to capture organizational members' shared beliefs in their joint abilities. Factor 3, 'prioritization and cosmopolitanism' which was also comprises of 5 items relates to internal and external institutional networks and priorities. Factor 4, comprises of 4 items, captures individual influencers of 'self-efficacy' including knowledge, skills, and time. Factor 5, 'financial resources', contains items related to internal and external budgets for KT activities.

Based on data collected during the stakeholder consultation process, these factors demonstrate face and content validity. That is, they appear to measure factors relevant to KT in these settings and represent the complex facets of the constructs

These five factors combined accounted for 69% of the total variance. The factor loadings, ranged from 0.40-0.77, are presented in **Table 4**; the intercorrelations between the factors ranged from 0.04 to 0.31. The final model observed a KMO measure of 0.554, and Bartlett's test of sphericity was significant ($\chi2$ (465) = 771.570, p < .000). Cronbach's alpha coefficients were 0.78, 0.73,

For peer review only - http://bmjopen.bmj.com/site/about/guidelines.xhtml

0.62, 0.68, and 0.52 respectively. Factors 1 and 2 report an alpha above 0.7 which is traditionally acceptable (34, 35). Though there are no firm bounds on Cronbach's alpha, factors 3-5 report a lower alpha which can indicate a need for cautious interpretation. However, given the theoretical underpinnings for each of these factors related both to concepts of individual and institutional readiness, and the small number of items loading to each factor, we concluded the Cronbach's alpha value are still helpful (36).

[Insert Table 4]

For peer review only

Table 4: Factor loadings, eigenvalues, and uniqueness

Survey questions	1	2	3	4	5	Eigenvalue	Uniqueness
Q96_8 Senior leadership/clinical management in my institution rewards innovation and creativity to improve KT.	0.5919					4.44856	0.6234
Q96_9 Financial incentives are available for me to conduct KT (e.g. bonus salary).	0.4766						0.4502
Q96_15 My institution provides trainings on knowledge translation activities.	0.5384						0.6646
Q96_17 Senior members/leadership of my institution provide me with connections to conduct KT.	0.7741						0.5035
Q96_18 People within my institution talk about their KT activities with each other.	0.6051						0.6781
Q98_2 Other faculty and staff members are available to collaborate on KT activities.	0.4296						0.5118
Q11_2 People at my institution are confident they can conduct KT activities.		0.5776				1.90881	0.6893
Q11_7 Others in my institution feel motivated to do KT.		0.7265					0.6405
Q97_5 The ministry relies on my institution more than other institutions to conduct KT.		0.4051					0.3678
Q97_10 Members of my government understand the importance of scientific data for making decisions about health.		0.4237					0.5058
Q98_15 When conducting KT activities, it is important to engage a wide range of stakeholders.		0.4826					0.5430
Q46 My institution includes KT in its strategic plan, mission, or vision.			0.6441			1.71858	0.7296
Q97_8 Ministry members and politicians in my country make health decisions without scientific consideration.			0.4222				0.6502
Q98_6 Senior members/Leadership of my institution use their networks to help others conduct KT.			0.4218				0.7574
Q98_12 I spend a lot of time planning my KT activities.			0.4568				0.6711
Q99_2 When I conduct KT activities, they address current priorities of the ministry.			0.5799				0.5658
Q94_5 I have received training to conduct KT activities.				0.4011		1.38363	0.6807
Q95_2 I have time to dedicate to KT in addition to my other tasks.				0.6935			0.6691
Q95_7 I know how to translate my data and key findings for policy makers.				0.4816			0.4893
Q98_1 KT teams at my institution have clearly defined roles and responsibilities.				0.4320			0.6713
Q97_12 Most projects I am involved with have budgeted for communications and advocacy activities.					0.4340	1.24604	0.6061
Q98_9 Conducting KT activities is more of an art than a science.					0.6443		0.5737
Q99_6 Financial resources are available at the ministry of health to support the cost of KT.					0.5578		0.6743

For peer review only - http://bmjopen.bmj.com/site/about/guidelines.xhtml

Values <0.4 are supressed. Factor 1 = “Institutional climate”; Factor 2 = “Organization change efficacy”; Factor 3 = “Prioritization and cosmopolitanism”; Factor 4 = “Self-efficacy”; Factor 5 = “Financial resources

For peer review only

DISCUSSION:

Assessing readiness for individuals and institutions in LMICs is different than in HICs. While some approaches, barriers, and facilitators are shared, low-resource settings have unique contexts that influence KT processes, the individuals who conduct KT, and the organizations in which they operate. We sought to develop a robust organizational readiness tool designed to reflect these contextual factors specifically for KT activities.

Five factors emerged as relevant for readiness to conduct KT in LMICs: Institutional Climate, Organization Change Efficacy, Prioritization and Cosmopolitanism, Self-efficacy, and Financial resources.

Institutional Climate: Institutional climate conceptualizes how individuals perceive and describe their work setting (37). This can include shared perceptions of what is rewarded within an institution and what is expected of people in their roles. In other words, organizational members' shared beliefs and values. The items included in this factor highlight different components of climate including rewards for innovation and creativity, financial incentives to conduct KT, and provision of trainings. Other items describe concepts of shared values through colleague collaboration, both in practice and availability to collaborate. Some interview participants described this as an enabling environment which could include access to infrastructure but also to networks:

> *An enabling environment is where you have everything you need then can be everything. In the ministry you have the internet, these laptops, these digital tools, the supportive director and supportive leadership who has recognized the importance of knowledge translation and is using it to influence change.*- Nigeria External 1

For peer review only - http://bmjopen.bmj.com/site/about/guidelines.xhtml

I think having mentors that consider knowledge translation important and that prioritize knowledge translation would be a great motivating factor. Mentors, as well as senior colleagues…but just seeing other people doing it, and seeing how they do it, and potentially how it can be rewarding, I think, is helpful and encouraging individual researchers to also conduct knowledge translation. – Nigeria Internal 2

Organization Change Efficacy: Weiner first described this concept as "organizational members' shared beliefs in their collective capabilities to organize and execute the courses of action involved in change implementation" (16). In 2014, Shea et al. assessed the psychometric properties of scales developed to measure change efficacy and other facets of organizational readiness (19). The change efficacy items focused on confidence in abilities to manage change processes, coordinate tasks, maintain momentum, and get investment. Items mapped to this factor from our scale similarly describe confidence and motivation (i.e. investment) and further contextualize this for KT. For example, items Q46, Q51, and Q69 account for the perceived role of external stakeholders, including the ministry of health and members of government, and their views on data and reliance on the institution, to conduct KT. Organization members may commit to change because they value it, have little choice, or feel obliged (16) – it makes sense that the views and actions of external stakeholders could influence those values, feelings, and obligations, particularly for health-related institutions.

Prioritization and Cosmopolitanism: Public health institutes are increasingly called on to align and adapt their activities to the health priorities of the country. Major international funders including the U.S. Centers for Disease Control have recently released calls for applications that address this issue of priority alignment between country governments and in-country public health organizations. When applied to academic organizations this tool highlighted the role of

For peer review only - http://bmjopen.bmj.com/site/about/guidelines.xhtml

institutional strategies, missions, and visions, and the importance of conducting KT activities that address national priorities. Integral to this concept is also the extent to which organizational networks can be leveraged to conduct KT. Bloland et al. argue that an important priority for public health institutions is to collaborate with MoHs to improve their abilities to not only accumulate data, but also manage that knowledge and translate it into actionable policies (38). This network component is fundamental to prioritization.

Self-efficacy: Readiness has often referred to an individual psychological state of motivation and plays an important role in many theories of behavior change including the Health Belief Model (e.g. self-efficacy) (39), Prochaska's Stages of Change Model (e.g. determination) (40), and Social Cognitive Theory (e.g. capability and self-efficacy) (41). It is unsurprising that individual level factors such as knowledge, training, and roles emerged as an important factor in this analysis. One unique item assessing time to dedicate to KT, included in this factor, was developed through data that emerged from the qualitative interviews. Participants reflected on competing priorities for their time and the need for protected and financially supported time to conduct KT.

> *We are researchers, and we are trained to do research and the research also takes up a lot of our time and energy. And knowledge translation itself takes a lot of time and energy and a completely different skill set; it's not a research skill.* – Indonesia Internal 1

Financial resources: KT activities, like most research and programmatic work, require the availability of financial resources. Items loading to this factor indicated that this is true not just for the academic institution (e.g. project budgets), but also at the MoH. KT models for evidence sharing frequently describe knowledge-push (knowledge supplied by researchers), demand-pull (demand for knowledge from policy-makers), and interactive approaches (42-44). These

For peer review only - http://bmjopen.bmj.com/site/about/guidelines.xhtml

underscore that KT is a dynamic process, requiring time, input, and resources from knowledge generators, translators, and users. Given the nuances of this process it is interesting but unsurprising that the item, "Conducting KT activities is more of an art than a science", loaded to this factor with a focus on resources.

Components of each of these five factors have been considered by existing organizational readiness tools though none have been combined in this way or used for KT specifically. This factor analysis builds on existing measurement tools and further demonstrates the dynamic nature of KT and underscores important contextual considerations for LMIC institutions. This includes the role of internal collaborations (institutional climate) and external networks (prioritization and cosmopolitanism), which often rely on leadership and senior institutional members. Literature has also noted the important role that funding organizations play in supporting KT in LMICs, both through a prioritization and a financial lens (45).

The final five-factor model captures many of the concepts represented in the original model but condenses constructs related to the individual (e.g. motivation and knowledge became self-efficacy) and the organization (e.g. different constructs of climate merged). Prioritization and cosmopolitanism, two constructs that capture internal and external networks, are consolidated in the final model, demonstrating the relationship between priority setting and networks. The outer setting and outer needs and resources, present across factors in the original model, are represented by organizational change efficacy and financial resources, highlighting how external factors can influence an organization's capacity to conduct KT. Other readiness domains not captured in the final model may not have been as relevant for the population studied across these settings but may still have relevance for other types of academic institutions in different LMICs.

For peer review only - http://bmjopen.bmj.com/site/about/guidelines.xhtml

This is the first organizational readiness tool designed for KT activities in LMICs. The model can be used by LMIC institutions to assess their readiness for KT, understand implementation challenges of KT initiatives, explore facilitators and barriers, and provide quantitative measurements for these institutions. The model can also be used to explore determinants of KT in these settings to inform the development of tailored strategies to improve institutional and individual capacity to conduct KT.

Strengths & Limitations: The development of this tool was rooted in organizational change theory, building on validated tools. We conducted the research across multiple contexts in both Africa and Asia to capture constructs generalizable to institutions based in LMICs. A significant limitation of this research is the small sample size. Most well-accepted guidelines for sample size to conduct EFA recommend a ratio of five to ten subjects per item. However, studies have argued smaller sample sizes may be justified when higher correlation coefficients are observed (46). The original tool was lengthy which could have resulted in response fatigue, leading to inaccurate data or incomplete responses. This may have been further exacerbated by selection bias if participants whose responses were dropped were similar in some way. Finally, we noted that participants from DRC and Ethiopia were possible outliers, skewing older (and therefore likely more experienced with KT) and more male than respondents from other countries while also having fewer responses than many of the other settings.

CONCLUSION:

KT is perceived as valuable for bridging the "know-do" gap, bringing evidence-based interventions into policies and practice. The five factors that emerged from this research as relevant to readiness to conduct KT highlight unique contextual influencers and opportunities for capacity strengthening in LMICs. The organizational focus of these factors further points to a

For peer review only - http://bmjopen.bmj.com/site/about/guidelines.xhtml

need for capacity building that includes but goes beyond individual training. Future research will be conducted to further understand the influencers of these readiness factors and systematically develop capacity building strategies for academic institutions in LMICs to conduct KT.

List of Abbreviations:

CFIR	Consolidated Framework for Implementation Research
EFA	Exploratory Factor Analysis
FA	Factor Analysis
HIC	High-Income Country
IQR	Inter-quartile range
KMO	Kaiser-Meyer-Olkin test
KT	Knowledge Translation
LMIC	Low-and Middle-Income Country
MoH	Ministry of Health
ORCA	Organizational Readiness to Change Assessment
ORIC	Organizational Readiness for Implementing Change
PIs	Principal Investigators
STRIPE	Synthesis and Translation of Research and Innovations from Polio Eradication
TCU ORC	Texas Christian University, Organizational Readiness to Change
TWG	Translating Evidence to Action Working Group

Declarations:

Ethics approval and consent to participate: This research was declared "Non-Human Subjects" by the Johns Hopkins Bloomberg School of Public Health Institutional Review Board on October 08, 2019. All participants were read a short statement on consent and provided a verbal agreement to prior to the interview.

Patient and Public Involvement: It was not appropriate or possible to involve the public in the design, or conduct, or reporting, or dissemination plans of our research.

Consent for publication: Not applicable

Availability of data and materials: The datasets used and/or analyzed during the current study are available from the corresponding author on reasonable request.

Competing interests: The authors declare that they have no competing interests

Funding: The parent-project to this research, STRIPE, is funded by the Bill and Melinda Gates Foundation. The Foundation played no role in funding this sub-set of research or in the design of the study and collection, analysis, and interpretation of the data, and in writing the manuscript.

For peer review only - http://bmjopen.bmj.com/site/about/guidelines.xhtml

Authors' contributions: AK conceived the study and paper with guidance from OOA. AK conducted the literature review, stakeholder engagement, tool development, tool distribution, and authored all drafts of the manuscript. AR supported data analysis and contributed to authorship of the initial draft of the paper. OOA provided significant oversight throughout the conceptualization, analysis, and write-up process and edited each draft. All authors read and approved the final manuscript.

Acknowledgements: The authors would like to acknowledge team members of the academic institutions included under the STRIPE academic consortium: Drs. Piyusha Majumdar, S.D. Gupta, and D.K. Mangal of the IIHMR University India, Drs. Riris Andono Ahmad, Yodi Mahendradhata, and Ms. Utsamani Cintyamena of Universitas Gadjah Mada Indonesia, Dr. Assefa Seme of Addis Ababa University Ethiopia, Drs. Eme Owoaje and Oluwaseun Akinyemi of the University of Ibadan Nigeria, Dr. Malabika Sarker of BRAC University Bangladesh, and Dr. Patrick Kayembe of the Kinshasa School of Public Health for their support of data collection. We would also like to thank the study participants for their time and contributions to the study.

REFERENCES

1. Evans D, Edejer T, Adams T, Lim S. Methods to assess the costs and health effects of interventions for improving health in developing countries. BMJ. 2005;331:1137-40.
2. Gwatkin D, Wagstaff A, Yazbeck A. Reaching the poor with health, nutrition, and population services: waht works, what doesn't and why. Geneva: World Bank; 2005.
3. Pantoja T, Opiyo N, Lewin S, Paulsen E, Ciapponi A, Wiysonge C, et al. Implementation strategies for health systems in low-income countries: an overview of systematic reviews. Cohcrane Dabatase of Systematic Reviews. 2017;9(9).
4. Straus S, Tetroe J, Graham I. Defining knowledge translation. Canadian Medicial Association Journal. 2009;181:165-68.
5. Rushmer R, Ward V, Nguyen T, Kuchenmuller T. Knowledge Translation: Key Concepts, Terms and Activities. Population Health Monitoring: Springer, Cham; 2019.
6. Bissell K, Viney K, Brostrom R, Gounder S, Khogali M, Kishore K. Building operational research capacity in the Pacific. Public Health Action. 2014;4.
7. Redman-MacLaren M, MacLaren D, Asugeni R, Fa'anuabae C, Harrington H, Muse A. "We can move forward": challenging historical inequity in public health research in Solomon Islands. International Journal of Equity in Health. 2010;9(25).
8. Bennett N, Cumberbatch C, Francis D. There are challenges in conducting systematic reviews in developing countries: the Jamaican experience. Journal of Clinical Epidemiology. 2015;68:1095-8.
9. Kalbarczyk A, Rodriguez D, Mahendradhata Y, Sarker M, Seme A, Majumdar P, et al. Barriers and facilitators to knowledge translation activities within academic institutions in low- and middle-income countries. Health Policy and Planning. 2021.
10. Gagliardi AR, Perrier L, Webster F, Leslie K, Bell M, Levinson W, et al. Exploring mentorship as a strategy to build capacity for knowledge translation research and practice: protocol for a qualitative study. Implementation Science. 2009;4(1):55.
11. Mallidou AA, Atherton P, Chan L, Frisch N, Glegg S, Scarrow G. Core knowledge translation competencies: a scoping review. BMC Health Services Research. 2018;18(1):502.
12. Brownson RC, Proctor EK, Luke DA, Baumann AA, Staub M, Brown MT, et al. Building capacity for dissemination and implementation research: one university's experience. Implementation Science. 2017;12(1):104.

For peer review only - http://bmjopen.bmj.com/site/about/guidelines.xhtml

13. Morrato EH, Rabin B, Proctor J, Cicutto LC, Battaglia CT, Lambert-Kerzner A, et al. Bringing it home: expanding the local reach of dissemination and implementation training via a university-based workshop. Implementation Science. 2015;10(1):94.

14. Proctor EK, Landsverk J, Baumann AA, Mittman BS, Aarons GA, Brownson RC, et al. The implementation research institute: training mental health implementation researchers in the United States. Implementation Science. 2013;8(1):105.

15. Murunga V, Oronje R, Bates I, Tagoe N, Pulford J. Review of published evidence on knowledge translation capacity, practice and support among researchers and research institutions in low- and middle-income countries. Health Research Policy and Systems. 2020;18(16).

16. Weiner B. A theory of organizational readiness for change. Implementation Science. 2009;4(67).

17. Solinis R. Are healthcare organizations ready for change? . International Journal of Health Policy and Management. 2018;7(12):1158-60.

18. Weiner B, Amick H, Lee S. Conceptualization and measurement of organizational readiness for change: a review of the literature in health services research and other fiedls. Med Care Res Rev. 2008;65(4):379-436.

19. Shea C, Jacobs S, Esserman D, Bruce K, Weiner B. Organizational readiness for implementing change: a psychometric assessment of a new measure. Implementation Science. 2014;9(7).

20. Gagnon M-P, Attieh R, Dunn S, Grandes G, Bully P, Estabrooks CA, et al. Development and Content Validation of a Transcultural Instrument to Assess Organizational Readiness for Knowledge Translation in Healthcare Organizations: The OR4KT. International Journal of Health Policy and Management. 2018;7(9):791-7.

21. Helfrich C, Li Y, Sharp N, Sales A. Organizational readiness to change assessment (ORCA): Development of an instrument based on the Promoting Action on Research in Health Services (PARiHS) framework. Implementation Science. 2009;4(38).

22. TCU Organizational Readiness for Change (ORC-D4). Forth Worth, TX Texas Christian University, Institute of Behavioral Research; 2009.

23. Gagnon MP, Attieh R, Ghandour el K, Légaré F, Ouimet M, Estabrooks CA, et al. A systematic review of instruments to assess organizational readiness for knowledge translation in health care. PLoS One. 2014;9(12):e114338.

24. Storkholm MH, Mazzocato P, Tessma MK, Savage C. Assessing the reliability and validity of the Danish version of Organizational Readiness for Implementing Change (ORIC). Implementation Science. 2018;13(1):78.

25. Bowles S, Louw J, Myers B. Perceptions of Organizational Functioning in Substance Abuse Treatment Facilities in South Africa. International Journal of Mental Health and Addiction. 2011;9(3):308-19.

26. Dearing J. Organizational readiness tools for global health intervention: A review. Frontiers in Public Health. 2018;6(56).

27. Alonge O, Neel A, Kalbarczyk A, Peters M, Mahendradhata Y, Sarker M, et al. Synthesis and translation of research and innovations from polio eradication (STRIPE): Initial findings from a global mixed methods study. BMC Public Health 2020.

28. Damschroder LJ, Aron DC, Keith RE, Kirsh SR, Alexander JA, JC L. Fostering implementation of health services research findings into practice: a consolidated framework for advancing implementation science. Implementation Science. 2009;4(50).

29. Means A, Kemp C, Gwayi-Chore M, Gimbel S, Soi C, Sherr K, et al. Evaluating and optimizing the consolidated framework for implementation research (CFIR) for use in low-and middle-income countries: a systematic review. Implementation Science. 2020;15(17).

30. Choi BC, Pak AW. A catalog of biases in questionnaires. Prev Chronic Dis. 2005;2(1):A13.

For peer review only

For peer review only - http://bmjopen.bmj.com/site/about/guidelines.xhtml

31. MacCallum R, Widaman K, Zhang S. Sample size in factor analysis Psychological Methods. 1999;4(1):84.
32. Arrindell W, Van der Ende J. An empirical test of the utility of the observations-to-variables ratio in factor and components analysis. Applied Psychological Measurement. 1985;9(2):165-78.
33. StataCorp. Stata Statistical Software: Release 13. College Station, TX: StataCorp LLC; 2013.
34. JA G, RR G. Calculating, interpreting, and reporting Cronbach's alpha reliability coefficient for Likert-type scales. Midwest Research to Practice Conference in Adult, Continuing, and Community Education; 2003.
35. JC N, IH B. Psychometric theory. New York: McGraw-Hill; 1994.
36. M T, R D. Making sense of Cronbach's alpha. International Journal of Medical Education. 2011;2:53-5.
37. Schneider B, Ehrhart M, Macey W. Organizational climate and culture. Annual Review of Psychology. 2013;64:361-88.
38. Bloland P, Simone P, Burkholder B, Slutsker L, De Cock K. The role of public health institutions in global health system strengthening efforts: The US CDC's Perspective. PLOS Medicine. 2012;9(4).
39. Strecher V, Champion V, Rosenstock I. The health belief model and health behavior. New York, NY, US: Plenum Press; 1997.
40. Prochaska J, Redding C, K E. The Transtheoretical Model and Stages of Change. In K, Glanz, BK Rimer & FM Lewis (Eds.) Health Behavior and Health Education: Theory, Research, and Pratice (3rd Ed.). San Francisco, CA: Jossey-Bass, Inc.; 2002.
41. Bandura A. Social foundations of thought and action: a social cognitive theory. Englewood Cliffs, NJ: Prentice-Hall; 1986.
42. Beyer H, Trice H. The utilization process: a conceptual framework and synthesis of empirical findings. Administrative Science Quarterly. 1982;27:591-622.
43. Huberman M. Steps toward an integrated model of research utilization. Knowledge. 1987;8:586-611.
44. Landry R, Amara N, Lamari M. Utilization of social science research knowledge in Canada. Research Policy. 2001;30:333-49.
45. Ezeh A, Lu J. Transforming the institutional landscape in sub-Saharan Africa: Considerations for leveraging Africa's research capacity to achieve socioeconomic development. Center for Global Development. 2019(147).
46. E G, WF V. Relation of sample size to the stability of component patterns. Psychological Bulletin. 1998;103(2):265-75.

For peer review only

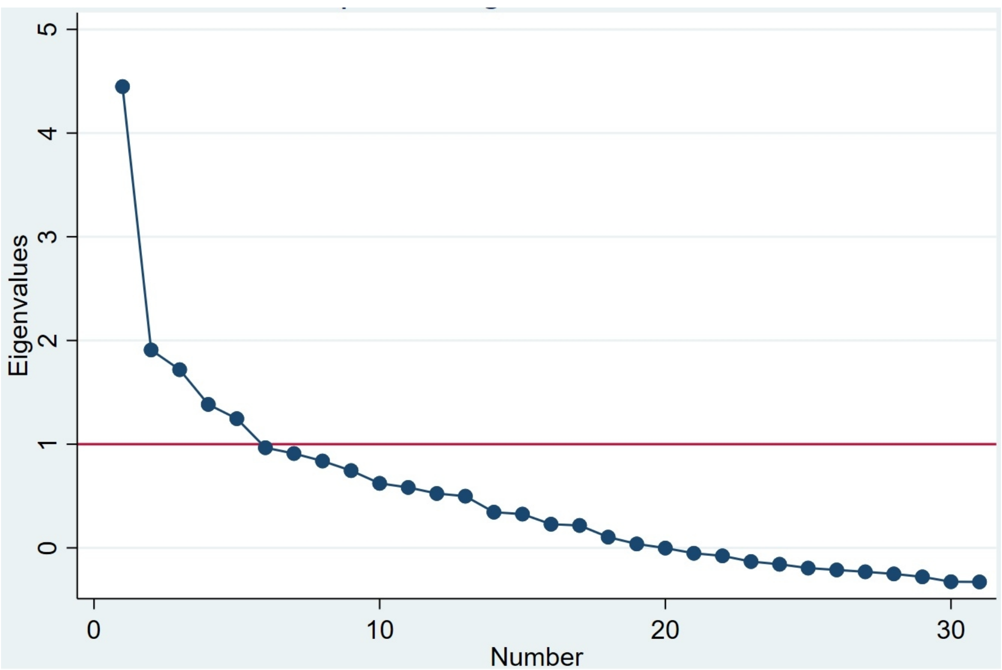

Final Factor Model Scree Plot

141x97mm (240 x 240 DPI)

Appendix I: Readiness Questionnaire

Assessing and Enhancing the Readiness of Academic Institutions in LMICs to Conduct Knowledge Translation Activities

In what country is your institution based?
What is your current age?
Which gender best identifies you:

Male

Female

Different identity

Prefer not to answer

What is your current professional focus? (Select all that apply)

Research

Administration

Leadership

Project Coordination

Communications

External Affairs

Development (e.g. Fundraising)

Management

Teaching

Finance

Information Technology

Regulatory Services (e.g. IRB)

Other (please describe) ____

Do you do any KT work related to priority health areas in your country?

Yes

No

Don't know

In your words, how do you define knowledge translation (KT)?

____

For peer review only

For peer review only - http://bmjopen.bmj.com/site/about/guidelines.xhtml

Have you ever conducted knowledge translation (KT)?

Yes

No

Unsure

Have you conducted any of the following activities in relation to health issues in the past 3-5 years? (select all that apply)

Written a policy brief or prepared briefing notes

Written an evidence summary

Conducted a stakeholder meeting

Conducted a policy dialogue

Engaged with an advocacy campaign

Engaged with policy makers to set priorities

Developed a short video for a policy maker

Engaged with the media (e.g. journalists)

Used a knowledge translation platform

Authored or co-authored an article published in a peer-review journal

Conducted a systematic and/or rapid review

Taught a course on communication, advocacy, stakeholder engagement, or KT

Worked with a journalist to disseminate information

Given a presentation at a scientific conference

Knowledge Translation is also referred to as "knowledge to policy/practice", "knowledge exchange" and "evidence to action". If you're less familiar with KT, consider these terms as you complete the survey.

For peer review only

Please identify the extent to which you agree or disagree with each of the following statements

	Strongly agree	Somewhat agree	Neither agree nor disagree	Somewhat disagree	Strongly disagree
I am confident that I can conduct KT activities.	○	○	○	○	○
People at my institution are confident they can conduct KT activities.	○	○	○	○	○
I feel personally motivated to do KT.	○	○	○	○	○
I feel personally motivated to do KT because I derive satisfaction and fulfillment.	○	○	○	○	○
I feel personally motivated to do KT because I receive rewards or benefits from my institution when I do.	○	○	○	○	○
I feel personally motivated to do KT because I will be punished by my institution if I do not.	○	○	○	○	○
Others in my institution feel motivated to do KT.	○	○	○	○	○

For peer review only

For peer review only - http://bmjopen.bmj.com/site/about/guidelines.xhtml

Please identify the extent to which you agree or disagree with each of the following statements

	Strongly agree	Somewhat agree	Neither agree nor disagree	Somewhat disagree	Strongly disagree
I know what KT is.	◯	◯	◯	◯	◯
I know how to do KT.	◯	◯	◯	◯	◯
I have the skills to conduct KT.	◯	◯	◯	◯	◯
I have experience conducting KT.	◯	◯	◯	◯	◯
I have received training to conduct KT activities.	◯	◯	◯	◯	◯
I frequently conduct KT activities.	◯	◯	◯	◯	◯

What health areas or health topics have been the focus of your KT activities in the past 3-5 years? Please list up to 5.

◯ Health Area/Topic 1 ________________________________

◯ Health Area/Topic 2 ________________________________

◯ Health Area/Topic 3 ________________________________

◯ Health Area/Topic 4 ________________________________

◯ Health Area/Topic 5 ________________________________

For peer review only

Please identify the extent to which you agree or disagree with each of the following statements:

	Strongly agree	Somewhat agree	Neither agree nor disagree	Somewhat disagree	Strongly disagree
I am able to conduct KT in addition to my other tasks at this institution.	○	○	○	○	○
I have time to dedicate to KT in addition to my other tasks.	○	○	○	○	○
I believe conducting KT is an important part of my job.	○	○	○	○	○
People in my institution should conduct KT.	○	○	○	○	○
I am passionate about conducting KT.	○	○	○	○	○
I feel personally rewarded when I conduct KT.	○	○	○	○	○
I know hot to translate my data and key findings for policy makers.	○	○	○	○	○
KT activities have a positive impact on the health of communities.	○	○	○	○	○
Doing KT activities improves the national/local relevance of my institution.	○	○	○	○	○

For peer review only

For peer review only - http://bmjopen.bmj.com/site/about/guidelines.xhtml

Please indicate the extent to which you agree or disagree with the following statements:

For peer review only

	Strongly agree	Somewhat agree	Neither agree nor disagree	Somewhat disagree	Strongly disagree
Senior leadership in my institution reward innovation and creativity in KT.	○	○	○	○	○
Senior Leadership in my institution provide staff with information on KT performance measures and guidelines.	○	○	○	○	○
In general in my institution, when there is agreement that KT needs to happen we have the necessary support in terms of budget or financial resources.	○	○	○	○	○
In general in my institution when there is agreement that KT needs to happen we have the necessary support in terms of training.	○	○	○	○	○
In general in my institution, when there is agreement that KT needs to happen we have the necessary support in terms of facilities.	○	○	○	○	○
In general in my institution when there is agreement that KT needs to happen we have the necessary support in terms of staffing.	○	○	○	○	○
Senior leadership management/staff opinion leaders at my institution set a high priority on the success of KT activities.	○	○	○	○	○
Senior leadership/clinical management in my institution reward innovation and creativity to improve KT.	○	○	○	○	○
Financial incentives are available for me to conduct KT (e.g. bonus salary).	○	○	○	○	○

For peer review only

My institution provides opportunities for professional development in KT (e.g. national and international conference support).	○	○	○	○	○
My institution cares about improving health services for communities in my country.	○	○	○	○	○
My colleagues believe it is important to translate our findings for policy makers and ministry members.	○	○	○	○	○
Members of leadership believe it is important for me and my colleagues to translate findings for policy makers and ministry members.	○	○	○	○	○
Conducting knowledge translation is an important consideration for promotion in my institution.	○	○	○	○	○
My institution provides trainings on knowledge translation activities.	○	○	○	○	○
If I want to conduct a KT activity, I know where to find people in my institution who can help.	○	○	○	○	○
Senior members/leadership of my institution provide me with connections to conduct KT.	○	○	○	○	○
People within my institution talk about their KT activities with each other.	○	○	○	○	○

For peer review only

My institution includes KT in its strategic plan, mission, or vision.

Strongly agree

Somewhat agree

Neither agree nor disagree

Somewhat disagree

Strongly disagree

If you indicated strongly agree or moderately/fairly agree to the question above, what health issues are prioritized by your institution in its strategic plan, mission or vision? (list up to 3)

- ◯ Priority Health Issue 1 ______________________________
- ◯ Priority Health Issue 2 ______________________________
- ◯ Priority Health Issue 3 ______________________________

For peer review only

Please indicate the extent to which you agree or disagree with the following statements:

For peer review only

	Strongly agree	Somewhat agree	Neither agree nor disagree	Somewhat disagree	Strongly disagree
My institution is well connected to the ministry of health.	◯	◯	◯	◯	◯
My institution determines research priorities in collaboration with the ministry of health.	◯	◯	◯	◯	◯
If my institution does not conduct KT with the ministry, another college or university in my country will.	◯	◯	◯	◯	◯
The funding organizations that support my research require KT activities.	◯	◯	◯	◯	◯
The ministry relies on my institution more than other institutions to conduct KT.	◯	◯	◯	◯	◯
Other institutions do more KT than my institution	◯	◯	◯	◯	◯
Donors support KT activities because they want to have a real impact on health.	◯	◯	◯	◯	◯
Ministry members and politicians in my country make health decisions without scientific consideration.	◯	◯	◯	◯	◯
If ministry members demand my institution conducts KT activities it is because a donor or funder requires them to do so.	◯	◯	◯	◯	◯
Members of my government understand the importance of scientific data for making decisions about health.	◯	◯	◯	◯	◯
Members of my government want to work with my institution to improve health.	◯	◯	◯	◯	◯
Most projects I am involved with have budgeted for communications and advocacy activities.	◯	◯	◯	◯	◯

For peer review only

Ministry members in my country prefer policy briefs to other forms of KT activities.	○	○	○	○	○

For peer review only

Please indicate the extent to which you agree or disagree with the following statements:

For peer review only

For peer review only - http://bmjopen.bmj.com/site/about/guidelines.xhtml

	Strongly agree	Somewhat agree	Neither agree nor disagree	Somewhat disagree	Strongly disagree
KT teams at my institution have clearly defined roles and responsibilities.	○	○	○	○	○
Other faculty and staff members are available to collaborate on KT activities.	○	○	○	○	○
I have regular opportunities to meet with ministry members to conduct KT.	○	○	○	○	○
Junior members of my team have opportunities to meet with ministry members to conduct KT.	○	○	○	○	○
I have at least one mentor who conducts KT with the ministry of health.	○	○	○	○	○
Senior members/Leadership of my institution use their networks to help others conduct KT.	○	○	○	○	○
I have time to regularly meet with members of the ministry to conduct KT activities.	○	○	○	○	○
I target my KT activities to different groups depending on the topic and who is involved.	○	○	○	○	○
Conducting KT activities is more of an art than a science.	○	○	○	○	○
My context plays a role in determining which KT activities I conduct.	○	○	○	○	○
Who I conduct KT activities with is as important as how I conduct the activities.	○	○	○	○	○
I spend a lot of time planning my KT activities.	○	○	○	○	○

For peer review only

For peer review only - http://bmjopen.bmj.com/site/about/guidelines.xhtml

It is important to regularly meet with stakeholders when conducting KT activities.	○	○	○	○	○
KT activities conducted by my institution tend to be performed ad-hoc.	○	○	○	○	○
When conducting KT activities, it is important to engage a wide range of stakeholders.	○	○	○	○	○

Please indicate the extent to which you agree or disagree with each of the following statements:

	Strongly agree	Somewhat agree	Neither agree nor disagree	Somewhat disagree	Strongly disagree
The ministry of health requests KT activities from my institution.	○	○	○	○	○
When I conduct KT activities, they address current priorities of the ministry.	○	○	○	○	○
Financial resources are available at my institution to support the cost of KT.	○	○	○	○	○
KT activities require more resources than are available at my institution.	○	○	○	○	○
The research I conduct is determined by the priorities of international donors.	○	○	○	○	○
Financial resources are available at the ministry of health to support the cost of KT.	○	○	○	○	○
I am aware of donors that fund KT activities.	○	○	○	○	○

For peer review only

In your opinion, which of the following are the top three **facilitators** of KT at your institution?

Dedicated personnel

Funding

Protected Time

Training

Institutional mission/vision/strategy

Relationships with ministry members

Institutional rewards

Motivated faculty, staff, and leadership

Other (please describe) ____

In your opinion, which of the following are the top three **barriers** of KT at your institution?

Funding

Training

Time

Networks with the Ministry of Health and other stakeholders

Awareness of KT

Interest from policy makers

Understanding the policy process

Experience with KT

Financial incentives or rewards to conduct KT

Leadership

Other (please describe) ____

For peer review only

For peer review only - http://bmjopen.bmj.com/site/about/guidelines.xhtml

Appendix II: Survey questions (v1) with Median [IQR]

**Survey questions**	**Median [IQR]**
Q1 I am confident that I can conduct KT activities	3 [2-4]
Q2 People at my institution are confident they can conduct KT activities.	2 [2-4]
Q5 I feel personally motivated to do KT because I receive rewards or benefits from my institution when I do.	2 [1-4]
Q6 I feel personally motivated to do KT because I will be punished by my institution if I do not.	5 [2-5]
Q7 Others in my institution feel motivated to do KT.	2 [1-2]
Q8 I know what KT is.	2 [2-4]
Q11 I have experience conducting KT.	2 [2-4]
Q12 I have received training to conduct KT activities.	3 [2-5]
Q13 I frequently conduct KT activities.	3 [2-4]
Q15 I have time to dedicate to KT in addition to my other tasks.	2 [2-4]
Q17 People in my institution should conduct KT.	3 [2-3]
Q18 I am passionate about conducting KT.	2 [2-4]
Q20 I know how to translate my data and key findings for policy makers.	2 [2-3]
Q21 KT activities have a positive impact on the health of communities.	4 [2-4]
Q23 Senior leadership in my institution reward innovation and creativity in KT.	2 [1-3]
Q26 In general in my institution when there is agreement that KT needs to happen we have the necessary support in terms of training.	2 [1-3]
Q30 Senior leadership/clinical management in my institution rewards innovation and creativity to improve KT.	2 [1-3]
Q31 Financial incentives are available for me to conduct KT (e.g. bonus salary).	3 [2-5]
Q32 My institution provides opportunities for professional development in KT (e.g. national and international conference support).	2 [2-4]
Q33 My institution cares about improving health services for communities in my country.	4 [2-4]
Q36 Conducting knowledge translation is an important consideration for promotion in my institution.	2 [2-4]
Q37 My institution provides trainings on knowledge translation activities.	3 [1-3]
Q38 If I want to conduct a KT activity, I know where to find people in my institution who can help.	2 [2-3]
Q39 Senior members/leadership of my institution provide me with connections to conduct KT.	2 [2-3]
Q40 People within my institution talk about their KT activities with each other.	2 [1-3]
Q41 My institution includes KT in its strategic plan, mission, or vision.	2 [1-4]
Q42 My institution is well connected to the ministry of health.	2 [2-4]
Q43 My institution determines research priorities in collaboration with the ministry of health.	2 [2-4]
Q44 If my institution does not conduct KT with the ministry, another college or university in my country will.	2 [1-3]
Q45 The funding organizations that support my research require KT activities.	2 [1-3]
Q46 The ministry relies on my institution more than other institutions to conduct KT.	2 [1-3]
Q47 Other institutions do more KT than my institution.	1.5 [1-3]
Q48 Donors support KT activities because they want to have a real impact on health.	2 [2-4]

For peer review only

Q49 Ministry members and politicians in my country make health decisions without scientific consideration.	2 [2-4]
Q50 If ministry members demand my institution conducts KT activities it is because a donor or funder requires them to do so.	2 [1-4]
Q51 Members of my government understand the importance of scientific data for making decisions about health.	2 [2-4]
Q52 Members of my government want to work with my institution to improve health.	2 [2-3]
Q53 Most projects I am involved with have budgeted for communications and advocacy activities.	2 [1-3.5]
Q54 Ministry members in my country prefer policy briefs to other forms of KT activities.	2 [1-3]
Q55 KT teams at my institution have clearly defined roles and responsibilities.	2 [1-3]
Q56 Other faculty and staff members are available to collaborate on KT activities.	2 [1-3]
Q59 I have at least one mentor who conducts KT with the ministry of health.	3 [2-4]
Q60 Senior members/Leadership of my institution use their networks to help others conduct KT.	2 [2-4]
Q61 I have time to regularly meet with members of the ministry to conduct KT activities.	3 [2-4]
Q62 I target my KT activities to different groups depending on the topic and who is involved.	2 [2-3]
Q63 Conducting KT activities is more of an art than a science.	2 [2-4]
Q64 My context plays a role in determining which KT activities I conduct.	2 [2-4]
Q65 Who I conduct KT activities with is as important as how I conduct the activities.	2 [2-4]
Q66 I spend a lot of time planning my KT activities.	2 [1-3]
Q67 It is important to regularly meet with stakeholders when conducting KT activities.	3 [2-4]
Q68 KT activities conducted by my institution tend to be performed ad-hoc.	2 [1-2]
Q69 When conducting KT activities, it is important to engage a wide range of stakeholders.	4 [2-4]
Q70 The ministry of health requests KT activities from my institution.	2 [1-3]
Q71 When I conduct KT activities, they address current priorities of the ministry.	2 [1-3]
Q72 Financial resources are available at my institution to support the cost of KT.	2 [1-3]
Q73 KT activities require more resources than are available at my institution.	2 [2-4]
Q74 The research I conduct is determined by the priorities of international donors.	2 [2-4]
Q75 Financial resources are available at the ministry of health to support the cost of KT.	2 [1-3]
Q76 I am aware of donors that fund KT activities.	2 [1-3]

For peer review only

BMJ Open

A mixed methods study to develop a tool to assess institutional readiness to conduct knowledge translation activities in low-and middle-income countries

Journal:	*BMJ Open*
Manuscript ID	bmjopen-2021-050049.R1
Article Type:	Original research
Date Submitted by the Author:	06-Sep-2021
Complete List of Authors:	Kalbarczyk, Anna; Johns Hopkins University Bloomberg School of Public Health, International Health<br>Rao, Aditi; Johns Hopkins University Bloomberg School of Public Health, International Health<br>Alonge, Olakunle; Johns Hopkins University Bloomberg School of Public Health
**Primary Subject Heading**:	Public health
Secondary Subject Heading:	Health policy
Keywords:	Health policy < HEALTH SERVICES ADMINISTRATION & MANAGEMENT, Organisational development < HEALTH SERVICES ADMINISTRATION & MANAGEMENT, PUBLIC HEALTH

SCHOLARONE™
Manuscripts

I, the Submitting Author has the right to grant and does grant on behalf of all authors of the Work (as defined in the below author licence), an exclusive licence and/or a non-exclusive licence for contributions from authors who are: i) UK Crown employees; ii) where BMJ has agreed a CC-BY licence shall apply, and/or iii) in accordance with the terms applicable for US Federal Government officers or employees acting as part of their official duties; on a worldwide, perpetual, irrevocable, royalty-free basis to BMJ Publishing Group Ltd ("BMJ") its licensees and where the relevant Journal is co-owned by BMJ to the co-owners of the Journal, to publish the Work in this journal and any other BMJ products and to exploit all rights, as set out in our licence.

The Submitting Author accepts and understands that any supply made under these terms is made by BMJ to the Submitting Author unless you are acting as an employee on behalf of your employer or a postgraduate student of an affiliated institution which is paying any applicable article publishing charge ("APC") for Open Access articles. Where the Submitting Author wishes to make the Work available on an Open Access basis (and intends to pay the relevant APC), the terms of reuse of such Open Access shall be governed by a Creative Commons licence – details of these licences and which Creative Commons licence will apply to this Work are set out in our licence referred to above.

Other than as permitted in any relevant BMJ Author's Self Archiving Policies, I confirm this Work has not been accepted for publication elsewhere, is not being considered for publication elsewhere and does not duplicate material already published. I confirm all authors consent to publication of this Work and authorise the granting of this licence.

For peer review only

Title: A mixed methods study to develop a tool to assess institutional readiness to conduct knowledge translation activities in low-and middle-income countries

Authors: Anna Kalbarczyk, Aditi Rao, Olakunle O. Alonge

Institutional Address for Authors:

Johns Hopkins Bloomberg School of Public Health

615 N Wolfe St, Baltimore, MD 21205

Corresponding Author:

Anna Kalbarczyk, DrPH, MPH

Johns Hopkins Bloomberg School of Public Health

615 N Wolfe St, Baltimore, MD 21205

Email: akalbarc@jhu.edu

Phone: +1 410 502 9873

For peer review only

ABSTRACT:

Objective: This paper describes the development of a tool for assessing organizational readiness to conduct KT among academic institutions in LMICs.

Design: A literature review and stakeholder consultation process was conducted to identify constructs relevant for assessing KT readiness in LMICs. These were face validated with LMIC stakeholders and organized into Likert scale questionnaire.

Participants: The questionnaire was distributed to researchers based at 6 LMIC academic institutions and members of a global knowledge-to-action thematic working group.

Outcome Measures: An exploratory factor analysis was used to identify underlying dimensions for assessing institutional readiness to conduct KT.

Results: 111 respondents with varied KT experiences from 10 LMICs were included in the analysis. We selected five factors and 23 items, with factor loadings from 0.40 to 0.77. These factors include 1) institutional climate, 2) organization change efficacy, 3) prioritization and cosmopolitanism, 4) self-efficacy, and 5) financial resources. These factors accounted for 69% of the total variance, with Cronbach's alpha coefficients of 0.78, 0.73, 0.62, 0.68, and 0.52, respectively.

Conclusions: This study identifies a tool for assessing readiness of LMIC academic institutions to conduct KT, and unique opportunities for building capacity. The organizational focus of these factors underscores the need for strategies that address organizational systems and structures in addition to individual skills. Future research will be conducted to understand determinants of these factors and develop a comprehensive set of capacity building strategies responsive to academic institutions in LMICs.

For peer review only - http://bmjopen.bmj.com/site/about/guidelines.xhtml

Keywords: knowledge translation, organizational readiness, factor analysis

Article Summary

Strengths and Limitations of this study

- The development of this tool was rooted in organizational change theory, building on validated tools.
- We conducted the research across multiple contexts in both Africa and Asia to capture constructs generalizable to institutions based in low-and middle-income countries.
- A significant limitation of this research is the small sample size. However, studies have argued smaller sample sizes may be justified when higher correlation coefficients are observed.
- The original tool was lengthy which could have resulted in response fatigue, leading to inaccurate data or incomplete responses.

For peer review only - http://bmjopen.bmj.com/site/about/guidelines.xhtml

INTRODUCTION:

Evidence-based, cost-effective interventions designed to improve health are available but under-utilized, particularly among the poorest populations in low-and middle-income countries (LMICs) (1, 2). A recent Cochrane review identified a wide range of strategies for implementing such interventions in LMICs (3). This gap between what we know through research and what is done in practice (i.e., 'know-do' gap), underscores the need for improved utilization of research by policy makers and practitioners in LMICs.

Knowledge Translation (KT) is one approach to addressing this gap that describes a process of research generation, synthesis, sharing, and utilization to improve health. Many definitions for KT exist and we use the one developed by the Canadian Institute of Health Research: "A dynamic and iterative process that includes the synthesis, dissemination, exchange, and ethically sound application of knowledge to improve health, provide more effective health services, and strengthen the health care system" (4). Examples of KT activities include evidence synthesis via policy briefs and systematic reviews, sharing knowledge through communities of practice, and forming large-scale partnerships and networks, which can be applied at different policy and practice levels to facilitate uptake of evidence and improve population health (5).

Academic institutions in LMICs are well-positioned to conduct KT activities as respected producers of research evidence and their ability to serve as knowledge brokers, but face a unique set of barriers driven by contextual factors including structural inequities (e.g. historically imbalanced relationships with institutions in the global north and research agendas driven by outside researchers) (6, 7) and limited resources (8). While some of these contextual factors (e.g., structural inequities) may be generalizable across most LMICs, others are specific to countries and may even vary within countries, e.g., the role of leadership, organizational structure and

For peer review only - http://bmjopen.bmj.com/site/about/guidelines.xhtml

culture, and the broader sociopolitical context. We conducted a qualitative study with participants in academic institutions in 6 countries (Bangladesh, Democratic Republic of the Congo (DRC), Ethiopia, India, Indonesia, and Nigeria) to identify barriers and determinants of institutional capacity to conduct KT activities across diverse LMICs contexts, and found that soft-skills (i.e. communication, self-awareness, and adaptability), robust networks, and alignment between institutional priorities and incentives are important factors that shape institutional capacity to conduct KT activities in LMICs (9). While strategies such as trainings, mentorship, and institutional leadership engagement have been developed to address these barriers and determinants (10, 11), these strategies have been mostly applied to academic institutions in high-income countries (HICs) (12-14) with limited empirical evidence of their effectiveness in LMIC settings (15). Irrespective of the strategies enacted to address barriers to conducting KT activities, academic institutions need to be self-aware on their role in KT, and readiness to undertake relevant KT activities addressing priority health issues to be successful (16).

Indeed, some institutions are becoming increasingly aware of the importance of organizational readiness and the need to implement processes that motivate and enhance change to facilitate the conduct of KT activities or address barriers to conducting KT activities (17, 18). Such processes could be motivated by increased demand from policy makers for evidence-based data or funders offering new opportunities that prioritize or require KT activities. Whereas specific KT activities (e.g., systematic review, establishing a community of practice) may vary for different academic institutions given differences in their priority health issue focus, policy cycle and resources – the similarities in the structure, function and objectives of academic institutions provide a conceptual basis for assessing their readiness to conduct different KT activities across different settings. Assessing organizational readiness enables exploration of facilitators and barriers for individuals

For peer review only - http://bmjopen.bmj.com/site/about/guidelines.xhtml

and organizations to implement capacity building strategies (19), and has been used to tailor approaches for addressing barriers to KT activities (20). However, most of these readiness assessments have been conducted in HICs, and there is an underuse of validated tools (18).

Validated readiness tools, used to assess institutional readiness for change (e.g. to implement any intervention, including KT activities), include Organizational Readiness for Implementing Change (ORIC) (19), Organizational Readiness to Change Assessment (ORCA) (21), and Texas Christian University Organizational Readiness to Change (TCU ORC) (22). These tools largely overlap and assess domains such as availability of resources, individual attributes and motivation, and organizational climate (19, 21, 22). Psychometric assessments have been conducted on these tools which largely support their reliability and structure (19, 23, 24). Most tools have been implemented in HICs, largely in health care settings including hospitals, primary care settings, public health agencies, and public sector organizations, and not specifically for KT. Gagnon et al. developed the OR4KT tool, designed to assess organizational readiness for KT in healthcare organizations, but they note that its applicability to LMICs may be limited given how different healthcare organizations are organized comparing HIC to LMIC settings (20). Our literature review identified only one validated tool, the TCU ORC, that had been used in a LMIC (25). This study utilized the TCU model to examine the organizational functioning of drug treatment facilities in South Africa but makes no mention of adapting the tool to address unique contextual factors. A recent systematic review identified 30 tools designed to address organizational readiness for institutions in LMICs but none were validated (26). The lack of readiness assessments specific to KT and availability of adapted tools for conducting readiness assessment further contributes to the 'know-do' gap in LMICs.

For peer review only - http://bmjopen.bmj.com/site/about/guidelines.xhtml

This paper describes an empirical study to develop a tool for assessing institutional readiness to conduct KT among academic institutions in 6 LMICs (Bangladesh, DRC, Ethiopia, India, Indonesia, and Nigeria). The paper builds on published tools for readiness assessments more broadly and developed for HIC settings and identifies constructs that are relevant for assessing readiness of academic institutions to do KT activities in LMICs, and the validity and reliability of an adapted tool measuring these constructs for LMIC contexts.

METHODS:

This research is embedded in a five-year parent-project, "Synthesis and Translation of Research and Innovations from Polio Eradication" (STRIPE), designed to map, synthesize, and disseminate knowledge from global efforts to eradicate polio (27). The STRIPE consortium is led by Johns Hopkins University in collaboration with 6 academic institutions and 1 research consultancy firm based in 7 LMICs. This research engaged the 6 academic collaborators, shown in **Table 1**, and was conducted in 3 sequential steps:

Table 1: STRIPE Academic Partners

Institution Name	Country
James P Grant School of Public Health, BRAC University	Bangladesh
School of Public Health, University of Kinshasa	Democratic Republic of the Congo (DRC)
College of Health Sciences, Addis Ababa University	Ethiopia
Indian Institute of Health Management Research University	India
Faculty of Medicine, Public Health, and Nursing, Gadjah Mada University	Indonesia
College of Medicine, University of Ibadan	Nigeria

Step 1: A Literature review was conducted to identify validated tools for measuring individual and institutional readiness. Standard search tools including PubMed and Google Scholar were searched in 2019 using combinations of the following terms: "tools", "organizational readiness",

For peer review only - http://bmjopen.bmj.com/site/about/guidelines.xhtml

and "institutional assessments". Thematic constructs and questionnaire items from the identified tools were extracted and organized by common domains. These were then mapped to the domains and constructs of the Consolidated Framework for Implementation Research (CFIR) which helped identify important gaps for implementation in LMICs. CFIR, a well-known framework, is comprised of five domains including intervention characteristics, outer setting, inner setting, individual characteristics, and implementation process (28). The framework has been widely used in LMICs as a practical structure for approaching complex programmatic and policy challenges (from planning to implementation to evaluation) such as conducting KT activities (29).

Institutional data was also collected from each academic partner including strategic plans, organograms, and information on recently published articles. Public information from each country's Ministry of Health (MoH) including recent reports and strategic plans were collected. Using the institutional data and information from the MoH, a content analysis was performed to establish health reference points for participants from each country (i.e. specific priority health issues on which the academic institution and ministry of health align).

Step 2: We conducted **stakeholder interviews** to evaluate the face and content validity of the constructs and items described from the review phase for LMIC contexts and explore new themes. The 18 stakeholders interviewed included representatives from each of the 6 academic STRIPE institutions in addition to their external government partners. Internal representatives met at least one of the following criteria: 1) Individuals involved in institutionally prioritized KT activities (health-issue specific), 2) Individuals involved in making strategic/policy decisions around KT activities at the institutional level, 3) Individuals in leadership that determine the internal context and external relationships of the institutions. External stakeholders included

For peer review only - http://bmjopen.bmj.com/site/about/guidelines.xhtml

policy makers who currently engaged or had been engaged with the academic institution in the past two years in efforts to conduct KT activities.

Inputs from this process focused largely on providing feedback on constructs of readiness as they related to less well-operationalized domains and constructs of CFIR in the literature (e.g. outer setting and characteristics of individuals). This process was also used to confirm health priorities in each country, identified initially in the literature review, and develop a list of KT activities commonly conducted across the settings. A draft tool for readiness assessment was then developed based on findings from the review and consultation steps. Country-level priorities and activities were incorporated throughout the tool to help illustrate complex KT items for participants.

Step 3: We developed a **quantitative survey** to assess the psychometric properties of the draft tool.

Tool design: The draft tool from step 2 was converted to a questionnaire using Microsoft Excel ©. Questions were grouped by demographics, the five CFIR domains, and barriers and facilitators to KT.

Demographic questions included country, gender, age, past experience with KT, and professional focus. Items designed to assess readiness were developed on a 5-point Likert scale ('strongly disagree to strongly agree'), following the design of the reviewed validated readiness tools. The final questions asked participants to select the top three barriers and facilitators to KT activities from a list generated based on steps 1 and 2. Negatively worded questions were reversed in numeric value, so the number 5 consistently reflected positive attitudes.

For peer review only - http://bmjopen.bmj.com/site/about/guidelines.xhtml

We followed guidelines outlined by Choi and Pak to avoid questionnaire biases (30). This included refraining from irrelevant redundancies, incorporating largely positively worded items, and utilizing priority references and activities to avoid ambiguity. Open-ended questions were minimized to reduce response fatigue and the format was designed to cluster Likert-scale questions, reducing the length of the questionnaire.

Survey population and procedure: The study population for the survey included faculty, staff, and leadership at each of the 6 academic STRIPE institutions in addition to members of the Translating Evidence to Action Working Group (TWG) within the Health Systems Global consortium who currently work for academic institutions based in LMICs. The TWG is a multidisciplinary group comprised of approximately 220 researchers, decision-makers, and implementers with a focus on the translation of health systems evidence into action (that is, KT) and supporting mechanisms to share best practices globally. STRIPE Principal Investigators (PI) at each institution identified faculty, staff, and leadership who had been involved in KT at their institution, amongst whom the survey was then distributed. The eligibility criteria for participating in the survey included: 1) must be an active member (e.g. staff, faculty, leadership) of an academic institution within the STRIPE consortium or TWG and 2) your institution must be based in a LMIC. A total of 200 eligible participants were identified via the STRIPE consortium - and were invited to participate in the survey via Qualtrics ©, an online survey software, with options to respond in English or French. TWG does not track demographics on its participants, so we do not know, of the 220 members, how many were eligible to complete the survey.

The survey questions were the same for all participants. However, the health priority reference points and list of KT activities identified for each institution in Step 2 was used to illustrate the

For peer review only - http://bmjopen.bmj.com/site/about/guidelines.xhtml

survey questions, and these differed from one academic institution to another. Members of the TWG, all highly familiar with KT, were not provided health priority references but were asked which health issues their institutions prioritized. The links to the Qualtrics surveys were circulated via email through STRIPE PIs, members of their leadership, or through local research assistants. Members of the thematic working group were contacted via email using the group's listserv. Survey data was collected from February 06, 2020 – March 25, 2020.

Responses were monitored for completeness (missing values) and correctness (invalid responses, outliers, and extreme observations). In-country liaisons at each STRIPE institution were asked to follow-up with potential respondents who may have started the survey but not completed it.

Analysis: We conducted Exploratory Factor Analysis (EFA) to identify underlying dimensions of institutional capacity and readiness to conduct KT activities among LMIC institutions. The process was iterative and exploratory, wherein each observed variable was considered as being a potential measure of factors, and the goal being to determine relationships between observed variables and factors that are strongest. EFA encompasses completing a series of sequential steps that involves evaluation of various options and making a decision at each step. These include assessing sample size adequacy, requisite correlation between variables, appropriate number of factors to include in the model, as well as selection of the factor rotation method.

We began with a sample size (N) to items (p) ratio of approximately 1.5:1. Guiding norms on an adequate sample size for conducting EFA state a desired ratio of at least 5:1 (31), while others agree smaller sample sizes can be justified when higher correlation coefficients are present (32). Adequacy of sample size was assessed via the Kaiser-Meyer-Olkin (KMO) test. The KMO statistic indicates the proportion of variance in the variables that might be cause by underlying factors. A score above 0.5 is generally regarded as acceptable. We proceeded with a Pearson

For peer review only - http://bmjopen.bmj.com/site/about/guidelines.xhtml

bivariate correlation of all items, inspecting for markedly high values, which indicate multicollinearity. Ultimately for pairs with correlation coefficients greater than 0.5, one item from each pair was removed based on a qualitative analysis of the variables. Factorability was confirmed by Bartlett's test of sphericity, which tests the null hypothesis that the original correlation matrix is an identity matrix, indicating that variables are unrelated and therefore unsuitable for structure detection. A significant value (<0.05) is requisite. Multicollinearity was explored via the determinant of the matrix, which should be greater than 0.00001.

The survey questions then underwent factor analysis (FA) with an oblique rotation (oblimin). We chose FA over principal component analysis, which assumes the total variance of the variables can be accounted for by means of its components (or factors), and hence that there is no error variance. Alternatively, FA assumes that the variables do not account for 100% of the variance, allowing it to be more flexible. Subsequently, a factor rotation allowed us to obtain a simpler, more interpretable solution for the FA. The choice of rotation depends on whether there is good theoretical reason to suppose the factors should be related or independent of each other. An oblique rotation allows a degree of correlation between the factors. In assessing the number of factors to retain, we considered multiple criteria. Factors with eigenvalues less than 1 were excluded, based on Guttman-Kaiser's rule. Using a Scree plot, all factors after the elbow were excluded. Only items that loaded highly (>0.5) and uniquely on each factor were retained. Factors with fewer than three variables loading on to it were also excluded. The proportion of the total variance explained by the retained factors was examined to be above the recommended minimum of 50%. Finally, Cronbach's alpha coefficient was calculated to test for scale reliability and internal consistency within and between factors. The norm is to accept values 0.7 or higher.

For peer review only - http://bmjopen.bmj.com/site/about/guidelines.xhtml

Data analysis was conducted in Stata version 13 (33).

This research was declared “Non-Human Subjects” by the Johns Hopkins Bloomberg School of Public Health Institutional Review Board on October 08, 2019.

RESULTS:

Literature Review

Review of the literature revealed 26 organizational readiness tools related to health institutions (including hospitals, clinics, research institutes, etc.) and 30 tools developed specifically to assess organizational readiness for implementing global health interventions; only 1 was designed specifically for KT. Both sets of tools had been collated and analyzed in systematic reviews (23, 26). Common constructs across these tools included availability of resources, individual attributes and motivation, and organizational climate.

Stakeholder Interviews

Eighteen stakeholders, both internal and external to the 6 STRIPE academic institution were interviewed. Three cross-cutting themes emerged as relevant for readiness to conduct KT in LMICs. This included 1) the complexity of the policy process and necessity of “soft-skills” (i.e., communication, self-awareness, relationship maintenance), 2) misalignment between institutional missions and incentives and 3) the role of internal and external networks. The results from the consultation process are described in more detail elsewhere (9). The constructs identified from the literature review and the cross-cutting themes from the consultation process were organized into a quantitative tool with 5 domains and 76 items, with new items developed for the themes. A total of 9 additional questions on demographics, facilitators, and barriers to doing KT were added to translate the tool into a survey questionnaire [Appendix 1].

For peer review only - http://bmjopen.bmj.com/site/about/guidelines.xhtml

Survey

We received 158 responses to the survey across the 6 STRIPE countries and TWG – 148 (74% of the eligible participants from the STRIPE countries responded to the survey). There were 47 respondents who completed 9% or less of the survey, which were subsequently dropped from analysis. These respondents did not cluster by country, age, or gender and appeared random. A total of 111 responses were included in the final analysis. According to Arrindell and van der Ende, a sample size (N) to items (p) ratio, i.e. N:p ratio of 3:1 is adequate for demonstrating a stable factor structure with an alpha level of 0.05 (32). Hence, our sample size of 111 is adequate for demonstrating the validity and reliability of a tool with at least 37 items.

There were 27 respondents from Bangladesh, 19 from Indonesia, 16 from India, 16 from Nigeria, 12 from DRC, 11 from Ethiopia, and 10 from the TWG representing other LMICs. Of the total 111 respondents, 53% (n=59) of respondents were male and 43% (n=48) were female. A majority of respondents (57%, n=64) were 30-49 years of age and most (59%, n=66) indicated they had experience conducting KT. Participants were asked to indicate their current professional focus/foci. Most respondents were engaged in research (83%, n=93) and teaching (62%, n=69). Other common foci included project coordination (36%, n=41), leadership (15%, n=17), and management (16%, n=18).

The most conducted KT activities included "taught a course on communication, advocacy, stakeholder engagement or KT" (98%, n=109), "conducted a stakeholder meeting" (62%, n=69), and "given a presentation at a scientific conference" (60%, n=67). Individuals who indicated having experience with KT activities, compared to those with no experience, were significantly more likely to have written a policy brief (p-value 0.0059), conducted a stakeholder meeting (p-value 0.0364), engaged with policy makers to set priorities (p-value 0.0129), and to have given a presentation at a scientific conference (p-value 0.0011). Two KT activities also varied significantly by country, "authored or co-authored an article in a peer-review journal" (p-value 0.0001), and "given a presentation at a scientific conference" (p-value 0.0002). Additional descriptive statistics are presented in **Table 2**.

For peer review only - http://bmjopen.bmj.com/site/about/guidelines.xhtml

1 **Table 2: Characteristics of Respondents**

Variable	Bangladesh n=27 (%)	DRC n=12 (%)	Ethiopia n=11 (%)	India n=16 (%)	Indonesia n=19 (%)	Nigeria n=16 (%)	Others n=10 (%)	Total n=111 (%)
**Age**								
18-29 years	13 (48.15)	0 (0.00)	0 (0.00)	0 (0.00)	6 (31.58)	0 (0.00)	0 (0.00)	19 (17.12)
30-49 years	12 (44.44)	6 (50.00)	8 (72.73)	7 (43.75)	11 (57.89)	12 (75.00)	8 (80.00)	64 (57.66)
50-69 years	2 (7.41)	5 (41.67)	3 (27.27)	7 (43.75)	0 (0.00)	3 (18.75)	2 (20.00)	22 (19.82)
≥70 years	0 (0.00)	1 (8.33)	0 (0.00)	0 (0.00)	0 (0.00)	0 (0.00)	0 (0.00)	1 (0.90)
**Gender**								
Male	16 (59.26)	6 (50.00)	8 (72.73)	10 (62.50)	5 (26.32)	9 (56.25)	5 (50.00)	59 (53.15)
Female	9 (33.33)	5 (41.67)	3 (27.27)	6 (37.50)	14 (73.68)	7 (43.75)	4 (40.00)	48 (43.24)
**Professional Focus**								
Research	22 (81.48)	12 (100.00)	10 (90.91)	14 (87.50)	14 (73.68)	13 (81.25)	8 (80.00)	93 (83.78)
Administration	1 (3.70)	1 (8.33)	0 (0.00)	1 (6.25)	1 (5.26)	5 (31.25)	0 (0.00)	9 (8.11)
Leadership	1 (3.70)	1 (8.33)	0 (0.00)	4 (25.00)	3 (15.79)	6 (37.50)	2 (20.00)	17 (15.32)
Project Coordination	6 (22.22)	6 (50.00)	1 (9.09)	12 (75.00)	8 (42.11)	5 (31.25)	3 (30.00)	41 (36.94)
Communications	1 (3.70)	0 (0.00)	0 (0.00)	3 (18.75)	1 (5.26)	1 (6.25)	2 (20.00)	8 (7.21)
External Affairs	0 (0.00)	2 (16.67)	0 (0.00)	0 (0.00)	1 (5.26)	0 (0.00)	0 (0.00)	3 (2.70)
Development	0 (0.00)	0 (0.00)	0 (0.00)	3 (18.75)	1 (5.26)	1 (6.25)	1 (10.00)	6 (5.41)
Management	4 (14.81)	1 (8.33)	0 (0.00)	6 (37.50)	3 (15.79)	3 (18.75)	1 (10.00)	18 (16.22)

For peer review only - http://bmjopen.bmj.com/site/about/guidelines.xhtml

For peer review only

Teaching	7 (25.93)	12 (100.00)	10 (90.91)	14 (87.50)	4 (21.05)	15 (93.75)	7 (70.00)	69 (62.16)
Finance	0 (0.00)	0 (0.00)	0 (0.00)	0 (0.00)	0 (0.00)	0 (0.00)	0 (0.00)	0 (0.00)
IT	0 (0.00)	0 (0.00)	0 (0.00)	1 (6.25)	0 (0.00)	0 (0.00)	0 (0.00)	1 (0.90)
Regulatory Services	2 (7.41)	1 (8.33)	0 (0.00)	1 (6.25)	0 (0.00)	0 (0.00)	1 (10.00)	5 (4.50)
**KT Experience**								
Yes	14 (51.85)	5 (41.67)	7 (63.64)	14 (87.50)	12 (63.16)	11 (68.75)	3 (30.00)	66 (59.46)
No	9 (33.33)	1 (8.33)	1 (9.09)	1 (6.25)	2 (10.53)	4 (25.00)	3 (30.00)	21 (18.92)
Unsure	4 (14.81)	6 (50.00)	3 (27.27)	1 (6.25)	4 (21.05)	1 (6.25)	3 (30.00)	22 (19.82)
**KT Activities**								
Written a policy brief	10 (37.04)	3 (25.000	2 (18.18)	8 (50.00)	11 (57.89)	7 (43.75)	3 (30.00)	44 (39.64)
Written an evidence summary	5 (18.52)	4 (33.33)	4 (36.36)	7 (43.75)	7 (36.84)	5 (31.25)	3 (30.00)	35 (31.53)
Conducted a stakeholder meeting	12 (44.44)	7 (58.33)	5 (45.45)	12 (75.00)	16 (84.21)	10 (62.50)	7 (70.00)	69 (62.16)
Conducted a policy dialogue	3 (11.11)	1 (8.33)	0 (0.00)	6 (37.50)	5 (26.32)	2 (12.50)	2 (20.00)	19 (17.12)
Engaged with an advocacy campaign	5 (18.52)	3 (25.00)	1 (0.09)	7 (43.75)	6 (31.58)	7 (43.75)	2 (20.00)	31 (27.93)
Engaged with policy makers to set priorities	3 (11.11)	4 (33.33)	3 (27.27)	6 (37.50)	9 (47.37)	5 (31.25)	4 (40.00)	34 (30.63)
Developed a video for a policy maker	5 (18.52)	1 (8.33)	0 (0.00)	4 (25.00)	4 (21.05)	2 (12.50)	1 (10.00)	17 (15.32)
Engaged with the media	5 (18.52)	3 (25.00)	2 (18.18)	8 (50.00)	5 (26.32)	8 (50.00)	5 (50.00)	36 (32.43)
Used a KT platform	4 (14.81)	4 (33.33)	2 (18.18)	7 (43.75)	4 (21.05)	0 (0.00)	3 (30.00)	24 (21.62)
Authored or co-authored	8 (29.63)	9 (75.00)	7 (63.64)	11 (68.75)	7 (36.84)	16 (100.00)	9 (90.00)	67 (60.36)
Conducted a systematic or rapid review	6 (22.22)	1 (8.33)	6 (54.55)	5 (31.25)	2 (10.53)	4 (25.00)	4 (40.00)	28 (25.23)
Taught a course on communication, advocacy, stakeholder engagement, or	27 (100.00)	12 (100.00)	11 (100.00)	14 (87.50)	19 (100.00)	16 (100.00)	10 (100.00)	109 (98.20)

For peer review only - http://bmjopen.bmj.com/site/about/guidelines.xhtml

KT								
Worked with a journalist to disseminate information	2 (7.41)	3 (25.00)	0 (0.00)	6 (37.50)	3 (15.79)	4 (25.00)	2 (20.00)	20 (18.02)
Given a presentation at a scientific conference	8 (29.63)	7 (58.33)	6 (54.55)	14 (87.50)	9 (47.37)	15 (93.75)	8 (80.00)	67 (60.36)

† Data were missing for some variables, therefore numbers do not always add to the total.

For peer review only

For peer review only - http://bmjopen.bmj.com/site/about/guidelines.xhtml

Exploratory factor analysis

We ran an exploratory factor analysis on the complete data set (version 0) which included 76 items. This approach yielded 22 factors; both the KMO measure and Barlett's test yielded no value. Constructs covered in these factors included individual motivation, organizational climate, organizational culture, internal resources, individual knowledge and skills, internal and external networks, funding sources, prioritization, and shared ethos for change (change valence). Many factors overlapped, each addressing similar or related constructs. The correlation matrix was reviewed for highly correlated items and those with correlations above 0.5 were dropped; 17 items were removed in this process. Highly correlated items included, "Q1: I am confident that I can conduct KT activities", "Q3: I feel personally motivated to do KT", and "Q10 I have the skills to conduct KT". Q10 was also heavily correlated with "Q9: I know how to do KT" and "Q11: I have experience conducting KT". Wherever possible, items were kept that did not correlate heavily with other items. These items were also reviewed to ensure they captured the same or similar information.

For the remaining 59 items (version 1.0) we repeated the EFA, followed by an oblique rotation, producing as simple a structure as possible while permitting correlations among factors. This yielded 17 factors, with a KMO measure of 0.4 and a significant value for Bartlett's test (p=0.000). The items, their medians, and inter-quartile range (IQR) for version 1.0 can be found in the supplemental material [Appendix 2]. Individual motivation, networks, prioritization, organizational climate, and resources were still captured by these factors. New constructs emerged in this model including institutional peer pressure, the process of conducting KT, and perceived value of KT.

For peer review only - http://bmjopen.bmj.com/site/about/guidelines.xhtml

All cross-loading items (i.e. items loading on more than one factor) or with a loading less than 0.5 were then dropped, resulting in the removal of 26 additional items (version 2.0). **Table 3** shows each item included in version 2.0, the item's median, and IQR. Some dropped items addressed individual motivation (e.g. Q13: I am passionate about conducting KT" and "KT activities have a positive impact on the health of communities") and institutional climate (e.g. Q32: My institution provides opportunities for professional development in KT"; Q59: I have at least one mentor who conducts KT with the ministry of health"). We re-ran the factor analysis with 31 items, followed by oblique rotation, and identified seven factors. The model produced a KMO of 0.52 and a significant value for Barlett's test (p=0.000). In this model one item (Q38: If I want to conduct a KT activity, I know where to find people in my institution who can help) loaded on two factors and was thus removed; in the resulting model, another item loaded to 2 factors and the item was dropped. A final set of 31 items was retained.

Table 3: Survey questions (v2) with Median [IQR]

**Survey questions**	**Median [IQR]**
Q26 In general in my institution when there is agreement that KT needs to happen we have the necessary support in terms of training.	3 [2-4]
Q30 Senior leadership/clinical management in my institution rewards innovation and creativity to improve KT.	2 [1-3]
Q31 Financial incentives are available for me to conduct KT (e.g. bonus salary).	3 [2-5]
Q37 My institution provides trainings on knowledge translation activities.	3 [1-3]
Q38 If I want to conduct a KT activity, I know where to find people in my institution who can help.	4 [3-4]
Q39 Senior members/leadership of my institution provide me with connections to conduct KT.	2 [2-3]
Q40 People within my institution talk about their KT activities with each other.	2 [1-3]
Q56 Other faculty and staff members are available to collaborate on KT activities.	2 [1-3]
Q2 People at my institution are confident they can conduct KT activities.	2 [2-4]
Q6 I feel personally motivated to do KT because I will be punished by my institution if I do not.	5 [3-5]
	2 [1-2]

For peer review only

For peer review only - http://bmjopen.bmj.com/site/about/guidelines.xhtml

Q7 Others in my institution feel motivated to do KT.	4 [3-4]
Q44 If my institution does not conduct KT with the ministry, another college or university in my country will.	4 [3-4]
Q45 The funding organizations that support my research require KT activities.	2 [1-3]
Q46 The ministry relies on my institution more than other institutions to conduct KT.	3 [2-3]
Q47 Other institutions do more KT than my institution.	2 [2-4]
Q51 Members of my government understand the importance of scientific data for making decisions about health.	4 [3-4]
Q52 Members of my government want to work with my institution to improve health.	4 [3-4]
Q54 Ministry members in my country prefer policy briefs to other forms of KT activities.	4 [2-4]
Q69 When conducting KT activities, it is important to engage a wide range of stakeholders.	2 [1-4]
Q41 My institution includes KT in its strategic plan, mission, or vision.	2 [2-4]
Q49 Ministry members and politicians in my country make health decisions without scientific consideration.	2 [2-4]
Q60 Senior members/Leadership of my institution use their networks to help others conduct KT.	2 [1-3]
Q66 I spend a lot of time planning my KT activities.	2 [1-3]
Q71 When I conduct KT activities, they address current priorities of the ministry.	3 [2-4]
Q76 I am aware of donors that fund KT activities.	4 [3-4]
Q11 I have experience conducting KT.	3 [2-5]
Q12 I have received training to conduct KT activities.	2 [2-4]
Q15 I have time to dedicate to KT in addition to my other tasks.	2 [2-3]
Q20 I know how to translate my data and key findings for policy makers.	2 [1-3]
Q55 KT teams at my institution have clearly defined roles and responsibilities.	2 [1-3.5]
Q53 Most projects I am involved with have budgeted for communications and advocacy activities.	2 [2-4]
Q63 Conducting KT activities is more of an art than a science.	2 [1-3]
Q75 Financial resources are available at the ministry of health to support the cost of KT.	

We re-ran the FA with the 31 retained items to identify a five-factor model. Items were selected based on the strength of the factor loading, uniqueness of the factors, the resulting scree-plot, and cross-loading criteria – and we selected 23 items for the five factors, with a N:p ratio of 5:1. **Figure 1** displays the eigen value plot of the final factor model. For the final five factor model, the average communality (aka uniqueness) of selected items was 0.61. The items (p) per factor (r) ratio (p:r ratio) in the final model presented in Table 3 is 23:5 with N = 111. According to

For peer review only - http://bmjopen.bmj.com/site/about/guidelines.xhtml

MacCallum et al., sample sizes of N = 100 and N = 200 are needed to estimate stable factor structure with 95% convergence for p:r ratio of 20:3 and 10:3 respectively if the average communality is low (less than 0.4) (31). If the communality is high (>0.4), as found in our study, a N = 60 is adequate for p:r ratio of 10:3 or 20:3 and will estimate the factor structure with over 99% convergence.

[Insert Figure 1]

The five final factors that emerged from the analysis were named 1) Institutional Climate, 2) Organization Change Efficacy, 3) Prioritization and Cosmopolitanism, 4) Self-efficacy, and 5) Financial resources, based on their item characteristics and the underlying theories for those items (9). Factor 1 contains 6 items and was labeled 'institutional climate' because each item described aspects of their institution, colleagues, and leadership. Factor 2 contains 5 items and was labeled 'organization change efficacy' to capture organizational members' shared beliefs in their joint abilities. Factor 3, 'prioritization and cosmopolitanism' which also comprises of 5 items relates to internal and external institutional networks and priorities. Factor 4, comprises of 4 items, captures individual influencers of 'self-efficacy' including knowledge, skills, and time. Factor 5, 'financial resources', contains items related to internal and external budgets for KT activities.

Based on data collected during the stakeholder consultation process, these factors demonstrate face and content validity. That is, they appear to measure factors relevant to KT in these settings and represent the complex facets of the constructs

These five factors combined accounted for 69% of the total variance. The factor loadings, ranged from 0.40-0.77, are presented in **Table 4**; the intercorrelations between the factors ranged from

For peer review only - http://bmjopen.bmj.com/site/about/guidelines.xhtml

0.04 to 0.31. The final model observed a KMO measure of 0.554, and Bartlett's test of sphericity was significant ($\chi2$ (465) = 771.570, $p < .000$). Cronbach's alpha coefficients were 0.78, 0.73, 0.62, 0.68, and 0.52 respectively. Factors 1 and 2 report an alpha above 0.7 which is traditionally acceptable (34, 35). Though there are no firm bounds on Cronbach's alpha, factors 3-5 report a lower alpha which can indicate a need for cautious interpretation. However, given the theoretical underpinnings for each of these factors related both to concepts of individual and institutional readiness, and the small number of items loading to each factor, we concluded the Cronbach's alpha value are still helpful (36).

[Insert Table 4]

For peer review only - http://bmjopen.bmj.com/site/about/guidelines.xhtml

For peer review only

For peer review only - http://bmjopen.bmj.com/site/about/guidelines.xhtml

Table 4: Factor loadings, eigenvalues, and uniqueness

Survey questions	1	2	3	4	5	Eigenvalue	Uniqueness
Q96_8 Senior leadership/clinical management in my institution rewards innovation and creativity to improve KT.	0.5919					4.44856	0.6234
Q96_9 Financial incentives are available for me to conduct KT (e.g. bonus salary).	0.4766						0.4502
Q96_15 My institution provides trainings on knowledge translation activities.	0.5384						0.6646
Q96_17 Senior members/leadership of my institution provide me with connections to conduct KT.	0.7741						0.5035
Q96_18 People within my institution talk about their KT activities with each other.	0.6051						0.6781
Q98_2 Other faculty and staff members are available to collaborate on KT activities.	0.4296					1.90881	0.5118
Q11_2 People at my institution are confident they can conduct KT activities.		0.5776					0.6893
Q11_7 Others in my institution feel motivated to do KT.		0.7265					0.6405
Q97_5 The ministry relies on my institution more than other institutions to conduct KT.		0.4051					0.3678
Q97_10 Members of my government understand the importance of scientific data for making decisions about health.		0.4237					0.5058
Q98_15 When conducting KT activities, it is important to engage a wide range of stakeholders.		0.4826					0.5430
Q46 My institution includes KT in its strategic plan, mission, or vision.			0.6441			1.71858	0.7296
Q97_8 Ministry members and politicians in my country make health decisions without scientific consideration.			0.4222				0.6502
Q98_6 Senior members/Leadership of my institution use their networks to help others conduct KT.			0.4218				0.7574
Q98_12 I spend a lot of time planning my KT activities.			0.4568				0.6711
Q99_2 When I conduct KT activities, they address current priorities of the ministry.			0.5799				0.5658
Q94_5 I have received training to conduct KT activities.				0.4011		1.38363	0.6807
Q95_2 I have time to dedicate to KT in addition to my other tasks.				0.6935			0.6691
Q95_7 I know how to translate my data and key findings for policy makers.				0.4816			0.4893
Q98_1 KT teams at my institution have clearly defined roles and responsibilities.				0.4320			0.6713
Q97_12 Most projects I am involved with have budgeted for communications and advocacy activities.					0.4340	1.24604	0.6061
Q98_9 Conducting KT activities is more of an art than a science.					0.6443		0.5737
Q99_6 Financial resources are available at the ministry of health to support the cost of KT.					0.5578		0.6743

For peer review only - http://bmjopen.bmj.com/site/about/guidelines.xhtml

Values <0.4 are supressed. Factor 1 = “Institutional climate”; Factor 2 = “Organization change efficacy”; Factor 3 = “Prioritization and cosmopolitanism”; Factor 4 = “Self-efficacy”; Factor 5 = “Financial resources

For peer review only

For peer review only - http://bmjopen.bmj.com/site/about/guidelines.xhtml

DISCUSSION:

Assessing readiness for individuals and institutions in LMICs is different than in HICs. While some approaches, barriers, and facilitators are shared, low-resource settings have unique contexts that influence KT processes, the individuals who conduct KT, and the organizations in which they operate. We sought to develop a robust organizational readiness tool designed to reflect these contextual factors specifically for KT activities. We know that while LMICs are diverse, certain barriers and determinants of institutional capacity to conduct KT are generalizable across diverse LMIC contexts (9) and evaluating readiness of academic institutions to conduct KT along these barriers and factors may provide a starting point for enacting KT practices in specific LMICs. However, like most KT and implementation research endeavors, such readiness assessments or any relevant tools will have to be further adapted to specific country contexts. The aim of this study is to provide those initial constructs that may be adaptable to different LMIC academic institutions for assessing their readiness to conduct KT activities.

Five factors emerged as relevant for readiness to conduct KT in LMICs: Institutional Climate, Organization Change Efficacy, Prioritization and Cosmopolitanism, Self-efficacy, and Financial resources.

Institutional Climate: Institutional climate conceptualizes how individuals perceive and describe their work setting (37). This can include shared perceptions of what is rewarded within an institution and what is expected of people in their roles. In other words, organizational members' shared beliefs and values. The items included in this factor highlight different components of climate including rewards for innovation and creativity, financial incentives to conduct KT, and provision of trainings. Other items describe concepts of shared values through colleague collaboration, both in practice and availability to collaborate. Some interview participants

For peer review only - http://bmjopen.bmj.com/site/about/guidelines.xhtml

described this as an enabling environment which could include access to infrastructure but also to networks:

An enabling environment is where you have everything you need then can be everything. In the ministry you have the internet, these laptops, these digital tools, the supportive director and supportive leadership who has recognized the importance of knowledge translation and is using it to influence change.- Nigeria External 1

I think having mentors that consider knowledge translation important and that prioritize knowledge translation would be a great motivating factor. Mentors, as well as senior colleagues…but just seeing other people doing it, and seeing how they do it, and potentially how it can be rewarding, I think, is helpful and encouraging individual researchers to also conduct knowledge translation. – Nigeria Internal 2

Organization Change Efficacy: Weiner first described this concept as "organizational members' shared beliefs in their collective capabilities to organize and execute the courses of action involved in change implementation" (16). In 2014, Shea et al. assessed the psychometric properties of scales developed to measure change efficacy and other facets of organizational readiness (19). The change efficacy items focused on confidence in abilities to manage change processes, coordinate tasks, maintain momentum, and get investment. Items mapped to this factor from our scale similarly describe confidence and motivation (i.e. investment) and further contextualize this for KT. For example, items Q46, Q51, and Q69 account for the perceived role of external stakeholders, including the ministry of health and members of government, and their views on data and reliance on the institution, to conduct KT. Organization members may commit to change because they value it, have little choice, or feel obliged (16) – it makes sense that the

For peer review only - http://bmjopen.bmj.com/site/about/guidelines.xhtml

views and actions of external stakeholders could influence those values, feelings, and obligations, particularly for health-related institutions.

Prioritization and Cosmopolitanism: Public health institutes are increasingly called on to align and adapt their activities to the health priorities of the country. Major international funders including the U.S. Centers for Disease Control have recently released calls for applications that address this issue of priority alignment between country governments and in-country public health organizations. When applied to academic organizations this tool highlighted the role of institutional strategies, missions, and visions, and the importance of conducting KT activities that address national priorities. Integral to this concept is also the extent to which organizational networks can be leveraged to conduct KT. Bloland et al. argue that an important priority for public health institutions is to collaborate with MoHs to improve their abilities to not only accumulate data, but also manage that knowledge and translate it into actionable policies (38). This network component is fundamental to prioritization.

Self-efficacy: Readiness has often referred to an individual psychological state of motivation and plays an important role in many theories of behavior change including the Health Belief Model (e.g. self-efficacy) (39), Prochaska's Stages of Change Model (e.g. determination) (40), and Social Cognitive Theory (e.g. capability and self-efficacy) (41). It is unsurprising that individual level factors such as knowledge, training, and roles emerged as an important factor in this analysis. One unique item assessing time to dedicate to KT, included in this factor, was developed through data that emerged from the qualitative interviews. Participants reflected on competing priorities for their time and the need for protected and financially supported time to conduct KT.

For peer review only - http://bmjopen.bmj.com/site/about/guidelines.xhtml

We are researchers, and we are trained to do research and the research also takes up a lot of our time and energy. And knowledge translation itself takes a lot of time and energy and a completely different skill set; it's not a research skill. – Indonesia Internal 1

Financial resources: KT activities, like most research and programmatic work, require the availability of financial resources. Items loading to this factor indicated that this is true not just for the academic institution (e.g. project budgets), but also at the MoH. KT models for evidence sharing frequently describe knowledge-push (knowledge supplied by researchers), demand-pull (demand for knowledge from policymakers), and interactive approaches (42-44). These underscore that KT is a dynamic process, requiring time, input, and resources from knowledge generators, translators, and users. Given the nuances of this process it is interesting but unsurprising that the item, "Conducting KT activities is more of an art than a science", loaded to this factor with a focus on resources.

Components of each of these five factors have been considered by existing organizational readiness tools though none have been combined in this way or used for KT specifically(23). This factor analysis builds on existing measurement tools and further demonstrates the dynamic nature of KT and underscores important contextual considerations for LMIC institutions. This includes the role of internal collaborations (institutional climate) and external networks (prioritization and cosmopolitanism), which often rely on leadership and senior institutional members. Literature has also noted the important role that funding organizations play in supporting KT in LMICs, both through a prioritization and a financial lens (45).

The final five-factor model captures many of the concepts represented in the original model but condenses constructs related to the individual (e.g. motivation and knowledge became self-efficacy) and the organization (e.g. different constructs of climate merged). Prioritization and

For peer review only - http://bmjopen.bmj.com/site/about/guidelines.xhtml

cosmopolitanism, two constructs that capture internal and external networks, are consolidated in the final model, demonstrating the relationship between priority setting and networks. The outer setting and outer needs and resources, present across factors in the original model, are represented by organizational change efficacy and financial resources, highlighting how external factors can influence an organization's capacity to conduct KT. Other readiness domains not captured in the final model may not have been as relevant for the population studied across these settings but may still have relevance for other types of academic institutions in different LMICs.

This is the first organizational readiness tool designed for KT activities in LMICs. The model can be used as a starting point and adapted by LMIC institutions to assess their readiness for KT, understand implementation challenges of KT initiatives, explore facilitators and barriers, and provide quantitative measurements for these institutions. The model can also be used to explore determinants of KT in these settings to inform the development of tailored strategies to improve institutional and individual capacity to conduct KT.

Strengths & Limitations: The development of this tool was rooted in organizational change theory, building on validated tools. We conducted the research across multiple contexts in both Africa and Asia to capture constructs generalizable to institutions based in LMICs. A significant limitation of this research is the small sample size. Most well-accepted guidelines for sample size to conduct EFA recommend a ratio of five to ten subjects per item. However, studies have argued smaller sample sizes may be justified when higher correlation coefficients are observed (46). The original tool was lengthy which could have resulted in response fatigue, leading to inaccurate data or incomplete responses. This may have been further exacerbated by selection bias if participants whose responses were dropped were similar in some way. Finally, we noted that participants from DRC and Ethiopia were possible outliers, skewing older (and therefore

For peer review only - http://bmjopen.bmj.com/site/about/guidelines.xhtml

likely more experienced with KT) and more male than respondents from other countries while also having fewer responses than many of the other settings.

CONCLUSION:

KT is perceived as valuable for bridging the "know-do" gap, bringing evidence-based interventions into policies and practice. The five factors that emerged from this research as relevant to readiness to conduct KT highlight unique contextual influencers and opportunities for capacity strengthening in LMICs. The organizational focus of these factors further points to a need for capacity building that includes but goes beyond individual training. Future research will be conducted to further understand the influencers of these readiness factors and systematically develop capacity building strategies for academic institutions in LMICs to conduct KT.

List of Abbreviations:

CFIR	Consolidated Framework for Implementation Research
EFA	Exploratory Factor Analysis
FA	Factor Analysis
HIC	High-Income Country
IQR	Inter-quartile range
KMO	Kaiser-Meyer-Olkin test
KT	Knowledge Translation
LMIC	Low-and Middle-Income Country
MoH	Ministry of Health
ORCA	Organizational Readiness to Change Assessment
ORIC	Organizational Readiness for Implementing Change
PIs	Principal Investigators
STRIPE	Synthesis and Translation of Research and Innovations from Polio Eradication
TCU ORC	Texas Christian University, Organizational Readiness to Change
TWG	Translating Evidence to Action Working Group

Figure Legend:

Figure 1: Final Factor Model Scree Plot

Declarations:

For peer review only - http://bmjopen.bmj.com/site/about/guidelines.xhtml

Ethics approval and consent to participate: This research was declared "Non-Human Subjects" by the Johns Hopkins Bloomberg School of Public Health Institutional Review Board on October 08, 2019. All participants were read a short statement on consent and provided a verbal agreement to prior to the interview.

Patient and Public Involvement: It was not appropriate or possible to involve the public in the design, or conduct, or reporting, or dissemination plans of our research.

Consent for publication: Not applicable

Availability of data and materials: The datasets used and/or analyzed during the current study are available from the corresponding author on reasonable request.

Competing interests: The authors declare that they have no competing interests

Funding: The parent-project to this research, STRIPE, is funded by the Bill and Melinda Gates Foundation (OPP1178578). The Foundation played no role in funding this sub-set of research or in the design of the study and collection, analysis, and interpretation of the data, and in writing the manuscript.

Authors' contributions: AK conceived the study and paper with guidance from OOA. AK conducted the literature review, stakeholder engagement, tool development, tool distribution, and authored all drafts of the manuscript. AR supported data analysis and contributed to authorship of the initial draft of the paper. OOA provided significant oversight throughout the conceptualization, analysis, and write-up process and edited each draft. All authors read and approved the final manuscript.

Acknowledgements: The authors would like to acknowledge team members of the academic institutions included under the STRIPE academic consortium: Drs. Piyusha Majumdar, S.D. Gupta, and D.K. Mangal of the IIHMR University India, Drs. Riris Andono Ahmad, Yodi Mahendradhata, and Ms. Utsamani Cintyamena of Universitas Gadjah Mada Indonesia, Dr. Assefa Seme of Addis Ababa University Ethiopia, Drs. Eme Owoaje and Oluwaseun Akinyemi of the University of Ibadan Nigeria, Dr. Malabika Sarker of BRAC University Bangladesh, and Dr. Patrick Kayembe of the Kinshasa School of Public Health for their support of data collection. We would also like to thank the study participants for their time and contributions to the study.

REFERENCES

1. Evans D, Edejer T, Adams T, Lim S. Methods to assess the costs and health effects of interventions for improving health in developing countries. BMJ. 2005;331:1137-40.
2. Gwatkin D, Wagstaff A, Yazbeck A. Reaching the poor with health, nutrition, and population services: waht works, what doesn't and why. Geneva: World Bank; 2005.
3. Pantoja T, Opiyo N, Lewin S, Paulsen E, Ciapponi A, Wiysonge C, et al. Implementation strategies for health systems in low-income countries: an overview of systematic reviews. Cohcrane Dabatase of Systematic Reviews. 2017;9(9).

4. Straus S, Tetroe J, Graham I. Defining knowledge translation. Canadian Medicial Association Journal. 2009;181:165-68.
5. Rushmer R, Ward V, Nguyen T, Kuchenmuller T. Knowledge Translation: Key Concepts, Terms and Activities. Population Health Monitoring: Springer, Cham; 2019.
6. Bissell K, Viney K, Brostrom R, Gounder S, Khogali M, Kishore K. Building operational research capacity in the Pacific. Public Health Action. 2014;4.
7. Redman-MacLaren M, MacLaren D, Asugeni R, Fa'anuabae C, Harrington H, Muse A. "We can move forward": challenging historical inequity in public health research in Solomon Islands. International Journal of Equity in Health. 2010;9(25).
8. Bennett N, Cumberbatch C, Francis D. There are challenges in conducting systematic reviews in developing countries: the Jamaican experience. Journal of Clinical Epidemiology. 2015;68:1095-8.
9. Kalbarczyk A, Rodriguez D, Mahendradhata Y, Sarker M, Seme A, Majumdar P, et al. Institutional facilitators and barriers for knowledge translation in low-and middle-income countries. Manuscript in preparataion. 2020.
10. Gagliardi AR, Perrier L, Webster F, Leslie K, Bell M, Levinson W, et al. Exploring mentorship as a strategy to build capacity for knowledge translation research and practice: protocol for a qualitative study. Implementation Science. 2009;4(1):55.
11. Mallidou AA, Atherton P, Chan L, Frisch N, Glegg S, Scarrow G. Core knowledge translation competencies: a scoping review. BMC Health Services Research. 2018;18(1):502.
12. Brownson RC, Proctor EK, Luke DA, Baumann AA, Staub M, Brown MT, et al. Building capacity for dissemination and implementation research: one university's experience. Implementation Science. 2017;12(1):104.
13. Morrato EH, Rabin B, Proctor J, Cicutto LC, Battaglia CT, Lambert-Kerzner A, et al. Bringing it home: expanding the local reach of dissemination and implementation training via a university-based workshop. Implementation Science. 2015;10(1):94.
14. Proctor EK, Landsverk J, Baumann AA, Mittman BS, Aarons GA, Brownson RC, et al. The implementation research institute: training mental health implementation researchers in the United States. Implementation Science. 2013;8(1):105.
15. Murunga V, Oronje R, Bates I, Tagoe N, Pulford J. Review of published evidence on knowledge translation capacity, practice and support among researchers and research institutions in low- and middle-income countries. Health Research Policy and Systems. 2020;18(16).
16. Weiner B. A theory of organizational readiness for change. Implementation Science. 2009;4(67).
17. Solinis R. Are healthcare organizations ready for change? . International Journal of Health Policy and Management. 2018;7(12):1158-60.
18. Weiner B, Amick H, Lee S. Conceptualization and measurement of organizational readiness for change: a review of the literature in health services research and other fiedls. Med Care Res Rev. 2008;65(4):379-436.
19. Shea C, Jacobs S, Esserman D, Bruce K, Weiner B. Organizational readiness for implementing change: a psychometric assessment of a new measure. Implementation Science. 2014;9(7).
20. Gagnon M-P, Attieh R, Dunn S, Grandes G, Bully P, Estabrooks CA, et al. Development and Content Validation of a Transcultural Instrument to Assess Organizational Readiness for Knowledge Translation in Healthcare Organizations: The OR4KT. International Journal of Health Policy and Management. 2018;7(9):791-7.
21. Helfrich C, Li Y, Sharp N, Sales A. Organizational readiness to change assessment (ORCA): Development of an instrument based on the Promoting Action on Research in Health Services (PARiHS) framework. Implementation Science. 2009;4(38).
22. TCU Organizational Readiness for Change (ORC-D4). Forth Worth, TX Texas Christian University, Institute of Behavioral Research; 2009.

For peer review only - http://bmjopen.bmj.com/site/about/guidelines.xhtml

23. Gagnon MP, Attieh R, Ghandour el K, Légaré F, Ouimet M, Estabrooks CA, et al. A systematic review of instruments to assess organizational readiness for knowledge translation in health care. PLoS One. 2014;9(12):e114338.
24. Storkholm MH, Mazzocato P, Tessma MK, Savage C. Assessing the reliability and validity of the Danish version of Organizational Readiness for Implementing Change (ORIC). Implementation Science. 2018;13(1):78.
25. Bowles S, Louw J, Myers B. Perceptions of Organizational Functioning in Substance Abuse Treatment Facilities in South Africa. International Journal of Mental Health and Addiction. 2011;9(3):308-19.
26. Dearing J. Organizational readiness tools for global health intervention: A review. Frontiers in Public Health. 2018;6(56).
27. Alonge O, Neel A, Kalbarczyk A, Peters M, Mahendradhata Y, Sarker M, et al. Synthesis and translation of research and innovations from polio eradication (STRIPE): Initial findings from a global mixed methods study. BMC Public Health 2020.
28. Damschroder LJ, Aron DC, Keith RE, Kirsh SR, Alexander JA, JC L. Fostering implementation of health services research findings into practice: a consolidated framework for advancing implementation science. Implementation Science. 2009;4(50).
29. Means A, Kemp C, Gwayi-Chore M, Gimbel S, Soi C, Sherr K, et al. Evaluating and optimizing the consolidated framework for implementation research (CFIR) for use in low-and middle-income countries: a systematic review. Implementation Science. 2020;15(17).
30. Choi BC, Pak AW. A catalog of biases in questionnaires. Prev Chronic Dis. 2005;2(1):A13.
31. MacCallum R, Widaman K, Zhang S. Sample size in factor analysis Psychological Methods. 1999;4(1):84.
32. Arrindell W, Van der Ende J. An empirical test of the utility of the observations-to-variables ratio in factor and components analysis. Applied Psychological Measurement. 1985;9(2):165-78.
33. StataCorp. Stata Statistical Software: Release 13. College Station, TX: StataCorp LLC; 2013.
34. JA G, RR G. Calculating, interpreting, and reporting Cronbach's alpha reliability coefficient for Likert-type scales. Midwest Research to Practice Conference in Adult, Continuing, and Community Education; 2003.
35. JC N, IH B. Psychometric theory. New York: McGraw-Hill; 1994.
36. M T, R D. Making sense of Cronbach's alpha. International Journal of Medical Education. 2011;2:53-5.
37. Schneider B, Ehrhart M, Macey W. Organizational climate and culture. Annual Review of Psychology. 2013;64:361-88.
38. Bloland P, Simone P, Burkholder B, Slutsker L, De Cock K. The role of public health institutions in global health system strengthening efforts: The US CDC's Perspective. PLOS Medicine. 2012;9(4).
39. Strecher V, Champion V, Rosenstock I. The health belief model and health behavior. New York, NY, US: Plenum Press; 1997.
40. Prochaska J, Redding C, K E. The Transtheoretical Model and Stages of Change. In K, Glanz, BK Rimer & FM Lewis (Eds.) Health Behavior and Health Education: Theory, Research, and Pratice (3rd Ed.). San Francisco, CA: Jossey-Bass, Inc.; 2002.
41. Bandura A. Social foundations of thought and action: a social cognitive theory. Englewood Cliffs, NJ: Prentice-Hall; 1986.
42. Beyer H, Trice H. The utilization process: a conceptual framework and synthesis of empirical findings. Administrative Science Quarterly. 1982;27:591-622.
43. Huberman M. Steps toward an integrated model of research utilization. Knowledge. 1987;8:586-611.

For peer review only - http://bmjopen.bmj.com/site/about/guidelines.xhtml

44. Landry R, Amara N, Lamari M. Utilization of social science research knowledge in Canada. Research Policy. 2001;30:333-49.
45. Ezeh A, Lu J. Transforming the institutional landscape in sub-Saharan Africa: Considerations for leveraging Africa's research capacity to achieve socioeconomic development. Center for Global Development. 2019(147).
46. E G, WF V. Relation of sample size to the stability of component patterns. Psychological Bulletin. 1998;103(2):265-75.

For peer review only

For peer review only - http://bmjopen.bmj.com/site/about/guidelines.xhtml

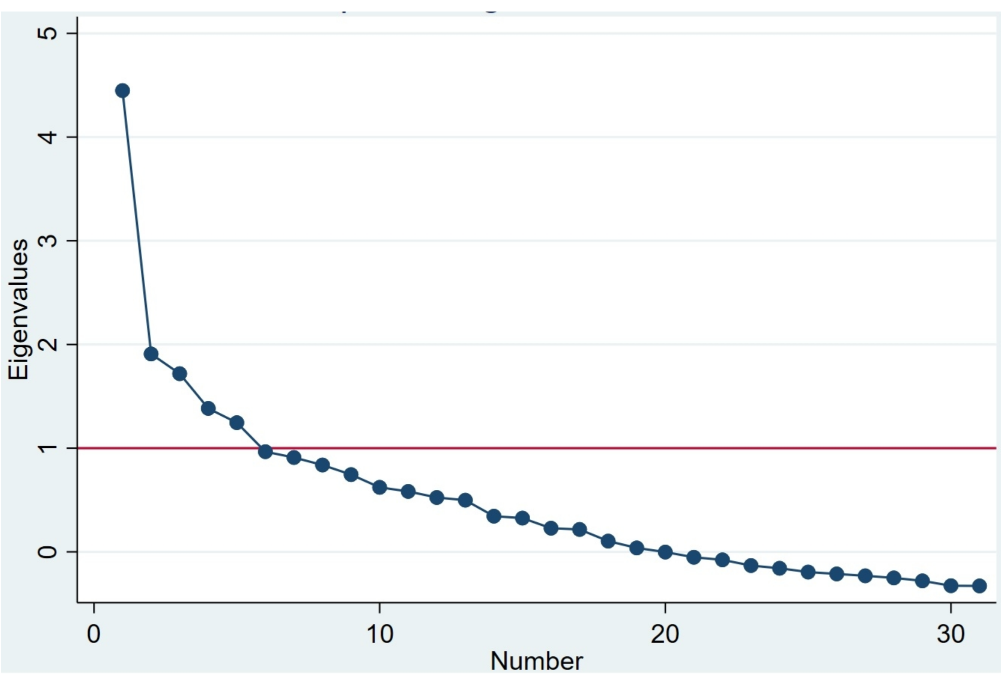

Final Factor Model Scree Plot

141x97mm (240 x 240 DPI)

Appendix I: Readiness Questionnaire

Assessing and Enhancing the Readiness of Academic Institutions in LMICs to Conduct Knowledge Translation Activities

In what country is your institution based?

What is your current age?

Which gender best identifies you:

- Male
- Female
- Different identity
- Prefer not to answer

What is your current professional focus? (Select all that apply)

- Research
- Administration
- Leadership
- Project Coordination
- Communications
- External Affairs
- Development (e.g. Fundraising)
- Management
- Teaching
- Finance
- Information Technology
- Regulatory Services (e.g. IRB)
- Other (please describe) ____

Do you do any KT work related to priority health areas in your country?

- Yes
- No
- Don't know

In your words, how do you define knowledge translation (KT)?

____

For peer review only

Have you ever conducted knowledge translation (KT)?

Yes

No

Unsure

Have you conducted any of the following activities in relation to health issues in the past 3-5 years? (select all that apply)

Written a policy brief or prepared briefing notes

Written an evidence summary

Conducted a stakeholder meeting

Conducted a policy dialogue

Engaged with an advocacy campaign

Engaged with policy makers to set priorities

Developed a short video for a policy maker

Engaged with the media (e.g. journalists)

Used a knowledge translation platform

Authored or co-authored an article published in a peer-review journal

Conducted a systematic and/or rapid review

Taught a course on communication, advocacy, stakeholder engagement, or KT

Worked with a journalist to disseminate information

Given a presentation at a scientific conference

Knowledge Translation is also referred to as "knowledge to policy/practice", "knowledge exchange" and "evidence to action". If you're less familiar with KT, consider these terms as you complete the survey.

For peer review only

Please identify the extent to which you agree or disagree with each of the following statements

	Strongly agree	Somewhat agree	Neither agree nor disagree	Somewhat disagree	Strongly disagree
I am confident that I can conduct KT activities.	○	○	○	○	○
People at my institution are confident they can conduct KT activities.	○	○	○	○	○
I feel personally motivated to do KT.	○	○	○	○	○
I feel personally motivated to do KT because I derive satisfaction and fulfillment.	○	○	○	○	○
I feel personally motivated to do KT because I receive rewards or benefits from my institution when I do.	○	○	○	○	○
I feel personally motivated to do KT because I will be punished by my institution if I do not.	○	○	○	○	○
Others in my institution feel motivated to do KT.	○	○	○	○	○

For peer review only

For peer review only - http://bmjopen.bmj.com/site/about/guidelines.xhtml

Please identify the extent to which you agree or disagree with each of the following statements

	Strongly agree	Somewhat agree	Neither agree nor disagree	Somewhat disagree	Strongly disagree
I know what KT is.	◯	◯	◯	◯	◯
I know how to do KT.	◯	◯	◯	◯	◯
I have the skills to conduct KT.	◯	◯	◯	◯	◯
I have experience conducting KT.	◯	◯	◯	◯	◯
I have received training to conduct KT activities.	◯	◯	◯	◯	◯
I frequently conduct KT activities.	◯	◯	◯	◯	◯

For peer review only

What health areas or health topics have been the focus of your KT activities in the past 3-5 years? Please list up to 5.

◯ Health Area/Topic 1 ____________________

◯ Health Area/Topic 2 ____________________

◯ Health Area/Topic 3 ____________________

◯ Health Area/Topic 4 ____________________

◯ Health Area/Topic 5 ____________________

For peer review only - http://bmjopen.bmj.com/site/about/guidelines.xhtml

Please identify the extent to which you agree or disagree with each of the following statements:

	Strongly agree	Somewhat agree	Neither agree nor disagree	Somewhat disagree	Strongly disagree
I am able to conduct KT in addition to my other tasks at this institution.	◯	◯	◯	◯	◯
I have time to dedicate to KT in addition to my other tasks.	◯	◯	◯	◯	◯
I believe conducting KT is an important part of my job.	◯	◯	◯	◯	◯
People in my institution should conduct KT.	◯	◯	◯	◯	◯
I am passionate about conducting KT.	◯	◯	◯	◯	◯
I feel personally rewarded when I conduct KT.	◯	◯	◯	◯	◯
I know hot to translate my data and key findings for policy makers.	◯	◯	◯	◯	◯
KT activities have a positive impact on the health of communities.	◯	◯	◯	◯	◯
Doing KT activities improves the national/local relevance of my institution.	◯	◯	◯	◯	◯

For peer review only

Please indicate the extent to which you agree or disagree with the following statements:

For peer review only

	Strongly agree	Somewhat agree	Neither agree nor disagree	Somewhat disagree	Strongly disagree
Senior leadership in my institution reward innovation and creativity in KT.	○	○	○	○	○
Senior Leadership in my institution provide staff with information on KT performance measures and guidelines.	○	○	○	○	○
In general in my institution, when there is agreement that KT needs to happen we have the necessary support in terms of budget or financial resources.	○	○	○	○	○
In general in my institution when there is agreement that KT needs to happen we have the necessary support in terms of training.	○	○	○	○	○
In general in my institution, when there is agreement that KT needs to happen we have the necessary support in terms of facilities.	○	○	○	○	○
In general in my institution when there is agreement that KT needs to happen we have the necessary support in terms of staffing.	○	○	○	○	○
Senior leadership management/staff opinion leaders at my institution set a high priority on the success of KT activities.	○	○	○	○	○
Senior leadership/clinical management in my institution reward innovation and creativity to improve KT.	○	○	○	○	○
Financial incentives are available for me to conduct KT (e.g. bonus salary).	○	○	○	○	○

For peer review only

For peer review only - http://bmjopen.bmj.com/site/about/guidelines.xhtml

My institution provides opportunities for professional development in KT (e.g. national and international conference support).	◯	◯	◯	◯	◯
My institution cares about improving health services for communities in my country.	◯	◯	◯	◯	◯
My colleagues believe it is important to translate our findings for policy makers and ministry members.	◯	◯	◯	◯	◯
Members of leadership believe it is important for me and my colleagues to translate findings for policy makers and ministry members.	◯	◯	◯	◯	◯
Conducting knowledge translation is an important consideration for promotion in my institution.	◯	◯	◯	◯	◯
My institution provides trainings on knowledge translation activities.	◯	◯	◯	◯	◯
If I want to conduct a KT activity, I know where to find people in my institution who can help.	◯	◯	◯	◯	◯
Senior members/leadership of my institution provide me with connections to conduct KT.	◯	◯	◯	◯	◯
People within my institution talk about their KT activities with each other.	◯	◯	◯	◯	◯

For peer review only

My institution includes KT in its strategic plan, mission, or vision.

- Strongly agree
- Somewhat agree
- Neither agree nor disagree
- Somewhat disagree
- Strongly disagree

If you indicated strongly agree or moderately/fairly agree to the question above, what health issues are prioritized by your institution in its strategic plan, mission or vision? (list up to 3)

- ◯ Priority Health Issue 1 ____
- ◯ Priority Health Issue 2 ____
- ◯ Priority Health Issue 3 ____

For peer review only

Please indicate the extent to which you agree or disagree with the following statements:

For peer review only

For peer review only - http://bmjopen.bmj.com/site/about/guidelines.xhtml

	Strongly agree	Somewhat agree	Neither agree nor disagree	Somewhat disagree	Strongly disagree
My institution is well connected to the ministry of health.	○	○	○	○	○
My institution determines research priorities in collaboration with the ministry of health.	○	○	○	○	○
If my institution does not conduct KT with the ministry, another college or university in my country will.	○	○	○	○	○
The funding organizations that support my research require KT activities.	○	○	○	○	○
The ministry relies on my institution more than other institutions to conduct KT.	○	○	○	○	○
Other institutions do more KT than my institution	○	○	○	○	○
Donors support KT activities because they want to have a real impact on health.	○	○	○	○	○
Ministry members and politicians in my country make health decisions without scientific consideration.	○	○	○	○	○
If ministry members demand my institution conducts KT activities it is because a donor or funder requires them to do so.	○	○	○	○	○
Members of my government understand the importance of scientific data for making decisions about health.	○	○	○	○	○
Members of my government want to work with my institution to improve health.	○	○	○	○	○
Most projects I am involved with have budgeted for communications and advocacy activities.	○	○	○	○	○

For peer review only

For peer review only - http://bmjopen.bmj.com/site/about/guidelines.xhtml

Ministry members in my country prefer policy briefs to other forms of KT activities.	○	○	○	○	○

For peer review only

Please indicate the extent to which you agree or disagree with the following statements:

For peer review only

	Strongly agree	Somewhat agree	Neither agree nor disagree	Somewhat disagree	Strongly disagree
KT teams at my institution have clearly defined roles and responsibilities.	○	○	○	○	○
Other faculty and staff members are available to collaborate on KT activities.	○	○	○	○	○
I have regular opportunities to meet with ministry members to conduct KT.	○	○	○	○	○
Junior members of my team have opportunities to meet with ministry members to conduct KT.	○	○	○	○	○
I have at least one mentor who conducts KT with the ministry of health.	○	○	○	○	○
Senior members/Leadership of my institution use their networks to help others conduct KT.	○	○	○	○	○
I have time to regularly meet with members of the ministry to conduct KT activities.	○	○	○	○	○
I target my KT activities to different groups depending on the topic and who is involved.	○	○	○	○	○
Conducting KT activities is more of an art than a science.	○	○	○	○	○
My context plays a role in determining which KT activities I conduct.	○	○	○	○	○
Who I conduct KT activities with is as important as how I conduct the activities.	○	○	○	○	○
I spend a lot of time planning my KT activities.	○	○	○	○	○

For peer review only

For peer review only - http://bmjopen.bmj.com/site/about/guidelines.xhtml

It is important to regularly meet with stakeholders when conducting KT activities.	○	○	○	○	○
KT activities conducted by my institution tend to be performed ad-hoc.	○	○	○	○	○
When conducting KT activities, it is important to engage a wide range of stakeholders.	○	○	○	○	○

Please indicate the extent to which you agree or disagree with each of the following statements:

	Strongly agree	Somewhat agree	Neither agree nor disagree	Somewhat disagree	Strongly disagree
The ministry of health requests KT activities from my institution.	○	○	○	○	○
When I conduct KT activities, they address current priorities of the ministry.	○	○	○	○	○
Financial resources are available at my institution to support the cost of KT.	○	○	○	○	○
KT activities require more resources than are available at my institution.	○	○	○	○	○
The research I conduct is determined by the priorities of international donors.	○	○	○	○	○
Financial resources are available at the ministry of health to support the cost of KT.	○	○	○	○	○
I am aware of donors that fund KT activities.	○	○	○	○	○

For peer review only

In your opinion, which of the following are the top three **facilitators** of KT at your institution?

Dedicated personnel

Funding

Protected Time

Training

Institutional mission/vision/strategy

Relationships with ministry members

Institutional rewards

Motivated faculty, staff, and leadership

Other (please describe) ____

In your opinion, which of the following are the top three **barriers** of KT at your institution?

Funding

Training

Time

Networks with the Ministry of Health and other stakeholders

Awareness of KT

Interest from policy makers

Understanding the policy process

Experience with KT

Financial incentives or rewards to conduct KT

Leadership

Other (please describe) ____

For peer review only

Appendix II: Survey questions (v1) with Median [IQR]

**Survey questions**	**Median [IQR]**
Q1 I am confident that I can conduct KT activities	3 [2-4]
Q2 People at my institution are confident they can conduct KT activities.	2 [2-4]
Q5 I feel personally motivated to do KT because I receive rewards or benefits from my institution when I do.	2 [1-4]
Q6 I feel personally motivated to do KT because I will be punished by my institution if I do not.	5 [2-5]
Q7 Others in my institution feel motivated to do KT.	2 [1-2]
Q8 I know what KT is.	2 [2-4]
Q11 I have experience conducting KT.	2 [2-4]
Q12 I have received training to conduct KT activities.	3 [2-5]
Q13 I frequently conduct KT activities.	3 [2-4]
Q15 I have time to dedicate to KT in addition to my other tasks.	2 [2-4]
Q17 People in my institution should conduct KT.	3 [2-3]
Q18 I am passionate about conducting KT.	2 [2-4]
Q20 I know how to translate my data and key findings for policy makers.	2 [2-3]
Q21 KT activities have a positive impact on the health of communities.	4 [2-4]
Q23 Senior leadership in my institution reward innovation and creativity in KT.	2 [1-3]
Q26 In general in my institution when there is agreement that KT needs to happen we have the necessary support in terms of training.	2 [1-3]
Q30 Senior leadership/clinical management in my institution rewards innovation and creativity to improve KT.	2 [1-3]
Q31 Financial incentives are available for me to conduct KT (e.g. bonus salary).	3 [2-5]
Q32 My institution provides opportunities for professional development in KT (e.g. national and international conference support).	2 [2-4]
Q33 My institution cares about improving health services for communities in my country.	4 [2-4]
Q36 Conducting knowledge translation is an important consideration for promotion in my institution.	2 [2-4]
Q37 My institution provides trainings on knowledge translation activities.	3 [1-3]
Q38 If I want to conduct a KT activity, I know where to find people in my institution who can help.	2 [2-3]
Q39 Senior members/leadership of my institution provide me with connections to conduct KT.	2 [2-3]
Q40 People within my institution talk about their KT activities with each other.	2 [1-3]
Q41 My institution includes KT in its strategic plan, mission, or vision.	2 [1-4]
Q42 My institution is well connected to the ministry of health.	2 [2-4]
Q43 My institution determines research priorities in collaboration with the ministry of health.	2 [2-4]
Q44 If my institution does not conduct KT with the ministry, another college or university in my country will.	2 [1-3]
Q45 The funding organizations that support my research require KT activities.	2 [1-3]
Q46 The ministry relies on my institution more than other institutions to conduct KT.	2 [1-3]
Q47 Other institutions do more KT than my institution.	1.5 [1-3]
Q48 Donors support KT activities because they want to have a real impact on health.	2 [2-4]

For peer review only

Q49 Ministry members and politicians in my country make health decisions without scientific consideration.	2 [2-4]
Q50 If ministry members demand my institution conducts KT activities it is because a donor or funder requires them to do so.	2 [1-4]
Q51 Members of my government understand the importance of scientific data for making decisions about health.	2 [2-4]
Q52 Members of my government want to work with my institution to improve health.	2 [2-3]
Q53 Most projects I am involved with have budgeted for communications and advocacy activities.	2 [1-3.5]
Q54 Ministry members in my country prefer policy briefs to other forms of KT activities.	2 [1-3]
Q55 KT teams at my institution have clearly defined roles and responsibilities.	2 [1-3]
Q56 Other faculty and staff members are available to collaborate on KT activities.	2 [1-3]
Q59 I have at least one mentor who conducts KT with the ministry of health.	3 [2-4]
Q60 Senior members/Leadership of my institution use their networks to help others conduct KT.	2 [2-4]
Q61 I have time to regularly meet with members of the ministry to conduct KT activities.	3 [2-4]
Q62 I target my KT activities to different groups depending on the topic and who is involved.	2 [2-3]
Q63 Conducting KT activities is more of an art than a science.	2 [2-4]
Q64 My context plays a role in determining which KT activities I conduct.	2 [2-4]
Q65 Who I conduct KT activities with is as important as how I conduct the activities.	2 [2-4]
Q66 I spend a lot of time planning my KT activities.	2 [1-3]
Q67 It is important to regularly meet with stakeholders when conducting KT activities.	3 [2-4]
Q68 KT activities conducted by my institution tend to be performed ad-hoc.	2 [1-2]
Q69 When conducting KT activities, it is important to engage a wide range of stakeholders.	4 [2-4]
Q70 The ministry of health requests KT activities from my institution.	2 [1-3]
Q71 When I conduct KT activities, they address current priorities of the ministry.	2 [1-3]
Q72 Financial resources are available at my institution to support the cost of KT.	2 [1-3]
Q73 KT activities require more resources than are available at my institution.	2 [2-4]
Q74 The research I conduct is determined by the priorities of international donors.	2 [2-4]
Q75 Financial resources are available at the ministry of health to support the cost of KT.	2 [1-3]
Q76 I am aware of donors that fund KT activities.	2 [1-3]

For peer review only
